# Organic Diradicals Bridged by Inverted Singlet–Triplet Units for Optical–Spin Interfaces

**DOI:** 10.1021/acs.jctc.5c01571

**Published:** 2025-11-29

**Authors:** Lorenzo Savi, Marco Tommaso Barreca, Matteo Bedogni, Francesco Di Maiolo

**Affiliations:** Department of Chemistry, Life Science and Environmental Sustainability, Università di Parma, 43124 Parma, Italy

## Abstract

Molecular platforms for optically addressable spin states are emerging as fascinating alternatives to solid-state spin centers, offering scalable synthesis, structural tunability, and chemical versatility. Here, we present a molecular design strategy for achieving photoinduced spin polarization in organic diradicals bridged by systems featuring an inverted singlet–triplet (InveST) energy gap. These InveST units possess HOMO and LUMO orbitals localized on complementary atomic sites. By covalently linking the non-SOMO-bearing positions of alternant hydrocarbon radicals to the LUMO-localized atoms of the InveST bridge, we construct diradicals in which the radical centers remain electronically decoupled in the ground state, yielding degenerate singlet and triplet configurations. Upon photoexcitation, the population of the InveST LUMO activates an excited-state exchange interaction between the radicals, generating a finite singlet–triplet gap and enabling spin-selective intersystem crossing to polarized triplet states. Using a combination of model Hamiltonians and multireference ab initio calculations, we establish design principles for tuning exchange interactions and spin–orbit coupling to achieve molecular-level control over optical–spin interfaces. The resulting InveST-bridged diradicals have emerged as promising scaffolds for molecular quantum technologies.

## Graphical abstract

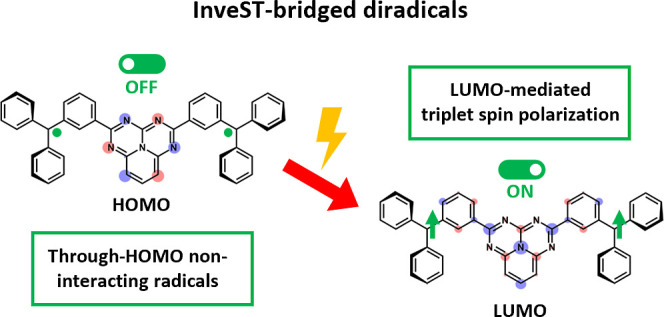

## 1 Introduction

Efforts to realize optically addressable electron spins have driven major advances in quantum information science, where the ability to initialize and read out spin states through optical transitions underpins optically detected magnetic resonance (ODMR) techniques. Current ODMR frameworks mainly rely on optically active spin defects in diamond, particularly nitrogen-vacancy (NV) centers, which offer exceptional spin coherence and robust photon–spin interfaces.^1–4^ However, these defect-based color centers face inherent limitations: they require postsynthetic incorporation with limited spatial control, constraining their scalability, reproducibility, and chemical tunability. Molecular spin systems offer a compelling alternative with advantages such as atomic-scale precision, modularity, and design-driven functionality that are difficult to achieve in solid-state platforms. Among these, organic diradicals have emerged as promising candidates for spin-active units.^5–17^ Their magnetic properties arise from the interplay of spin–spin interactions, frontier orbital topology, and excited-state dynamics, all of which can be systematically tailored through molecular design. Here, we introduce a molecular design strategy for a novel optical–spin interface that enables light-induced switching of spin–spin interactions in diradicals, where the two radical units are connected by a dye with an inverted singlet–triplet (InveST) energy gap (Figure 1a).

**Figure 1:**
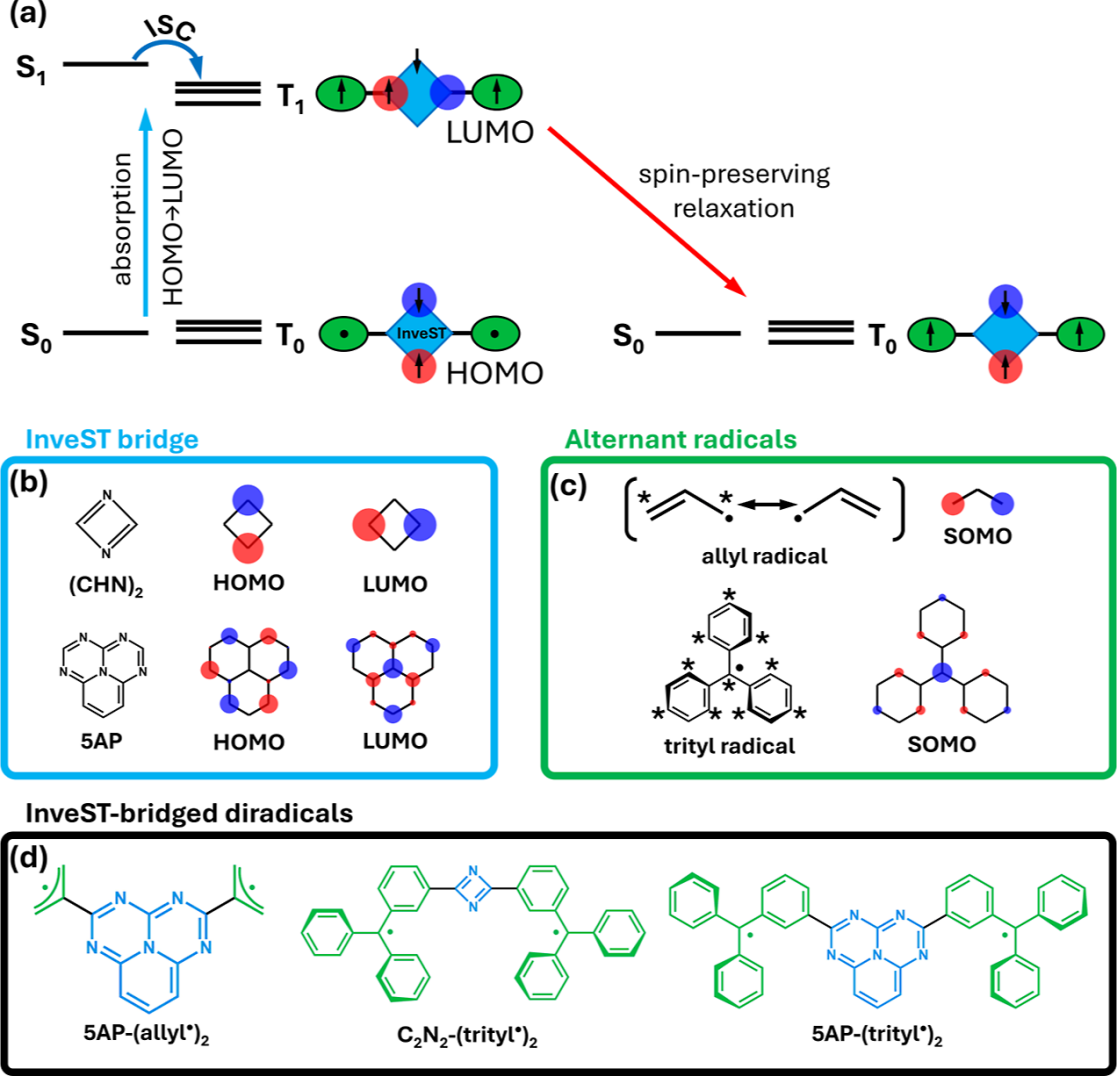
(a) Schematic representation of the InveST-bridged diradical design. The simplified Jablonski diagram shows the InveST-localized HOMO → LUMO transition that induces spin–spin locking in the excited state and its subsequent spin-preserving relaxation to the triplet ground state. (b) Molecular structures of two prototypical InveST bridges, namely, 1,3,4,6,9b-pentaazaphenalene (5AP) and 1,3-diazete, together with their frontier PPP Hartree–Fock MOs. (c) Molecular structures of two prototypical alternant radicals, namely, allyl and trityl radicals, together with their SOMO. (d) Molecular structures of the three prototypical InveST-bridged diradicals discussed in this work.

In InveST systems, the highest occupied molecular orbital (HOMO) and lowest unoccupied molecular orbital (LUMO) are localized on complementary atomic sites with minimal spatial overlap.^18,19^ As a result, the lowest excited state predominantly exhibits a multiresonant charge-transfer (MRCT) character, with the electron density shifting from the HOMO to the LUMO. Under these conditions, the exchange integral, which governs the singlet–triplet (ST) splitting, is very small, and the typically minor dynamic spin polarization correction—arising from doubly excited determinants involving HOMO, LUMO, and other π orbitals in the wave function expansion—can become significantly large to invert the ST energy gap.^20–25^ Such ST inversion has been experimentally observed in heptazine derivatives^26–32^ and 1,3,4,6,9b-pentaazaphenalene (5AP),^33,34^ both of which feature the HOMO and LUMO localized on distinct and complementary atomic sites (Figure 1b). Moreover, some of us have recently shown^35,36^ that this HOMO–LUMO complementarity is a general feature of polyenes with alternating electron-donor (D) and electron-acceptor (A) groups, with 1,3-diazete representing the smallest molecular motif that exhibits this behavior (see Figure 1b).

For the radical units, we employ alternant hydrocarbon radicals.^9–11,37–39^ In these systems, the carbon atoms can be partitioned into two distinct sets using a “starring” process: every other carbon atom is assigned a star such that no two starred atoms are directly bonded (see Figure 1c). This construction ensures that starred atoms bond only with unstarred atoms and vice versa. A hallmark feature of alternant hydrocarbon radicals is that their singly occupied molecular orbital (SOMO) is nonbonding and localized exclusively on one of the two sublattices, specifically the one containing more atoms (see Figure 1c).

By covalently linking the non-SOMO-bearing sites of each alternant hydrocarbon radical to the LUMO-localized positions of the InveST dye, spin–spin interactions can be systematically suppressed in the ground state, resulting in diradicals with intrinsic disjoint character (see Figure 1d). Each nonbonding SOMO is confined to one of the radical subunits, and the small interaction between these disjoint orbitals leads to nearly degenerate lowest-energy singlet and triplet states.^40,41^ Upon photoexcitation of the InveST bridge via a HOMO → LUMO transition, the LUMO becomes occupied and mediates interaction between the radicals, effectively switching exchange interactions in the excited state. Because the InveST bridge features a singlet–triplet inversion, this initial excitation is singlet in character, and the resulting exchange interaction with the radical spins drives the formation of an overall triplet excited state.

The optical–spin interface works through spin-selective interactions among excited states, enabling preferential population and decay pathways that distinguish between different spin configurations.^9–11^ In this context, intersystem crossing (ISC) mediated by spin–orbit coupling (SOC) plays a key role in facilitating transitions between singlet and triplet manifolds. SOC vanishes identically in perfectly planar π-conjugated systems; however, any deviation from planarity introduces the necessary mixing between different spin manifolds. The InveST-bridged diradicals in Figure 1d exhibit torsional flexibility around the two bonds connecting the InveST core to the alternating hydrocarbon radicals. At room temperature, these torsional degrees of freedom are thermally populated, generating a dynamic ensemble of conformations with varying dihedral angles between the bridge and the radical units. Thermal access to nonplanar conformations maintains active SOC at the connecting bonds, promoting ISC.

Using a combined theoretical approach based on the Pariser–Parr–Pople (PPP) model^9,35,42–46^ and high-level multireference ab initio calculations, we show that InveST-bridged diradicals are promising molecular alternatives to NV centers for quantum information applications. Our results show that SOC-mediated ISC can occur efficiently between the first excited singlet (S_1_) and triplet (T_1_) states, creating excited-state dynamics useful for ODMR mechanisms that enable ground-state triplet spin polarization. We validate this design strategy using two representative InveST bridges, 1,3-diazete and 5AP, covalently linked to allyl and triphenylmethyl (trityl) radical units (Figure 1d).

## 2 Results and Discussion

### 2.1 Electronic Structure Characterization

The PPP model is one of the simplest yet most effective frameworks for studying the electron correlation in π-conjugated molecules. Like the Hückel model, it considers only the 2p_*z*_ atomic orbitals that are perpendicular to the molecular plane at each atomic site. However, unlike the Hückel model, the PPP model includes electron–electron (e–e) interactions by adopting the zero differential overlap approximation, which neglects overlap integrals between 2p_*z*_ orbitals on different atoms. The PPP Hamiltonian, expressed in the real-space atomic orbital basis, reads

1 $$H_{PPP}=\sum_{\mu }\varepsilon_{\mu }n_{\mu }-\sum_{\mu \nu ,\nu >\mu }\sum_{\sigma }t_{\mu \nu }\left(a_{\mu \sigma }^{†}a_{\nu \sigma }+a_{\nu \sigma }^{†}a_{\mu \sigma }\right)+\sum_{\mu }U_{\mu }n_{\mu ↑}n_{\mu ↓}+\sum_{\mu \nu ,\nu >\mu }V_{\mu \nu }\left(Z_{\mu }-n_{\mu }\right)\left(Z_{\nu }-n_{\nu }\right)$$

where *a*_μσ_ and *a*_μσ_^†^ denote the annihilation and creation operators, respectively, for an electron with spin σ on atom μ, while the operator $n_{\mu }=\sum_{\sigma }a_{\mu \sigma }^{†}a_{\mu \sigma }$ gives the total electron occupancy at site μ. The terms in the first line of the Hamiltonian include ε_μ_, the energy of the 2p_*z*_ orbital centered on atom μ, and *t*_μν_, which is the hopping amplitude between neighboring atoms μ and ν. Within the PPP framework, hopping is limited to pairs of atoms connected via a σ bond. The second line introduces electron–electron repulsion terms: *U*_μ_ accounts for the on-site Coulomb interaction between two electrons occupying the same 2p_*z*_ orbital, while *V*_μν_ represents the intersite Coulomb repulsion between electrons on atoms μ and ν, as further discussed in the Supporting Information Section S1.1. The quantity *Z*_μ_ corresponds to the net nuclear charge at site μ after subtracting the π-electron contribution (*Z*_μ_ = 1 for both carbon and aza nitrogen atoms and *Z*_μ_ = 2 for pyrrole nitrogens).

The torsional flexibility around the bonds connecting the InveST core to the radical units plays a key role in modulating the electronic communication between the molecular fragments. While the PPP model was originally developed for planar π-conjugated hydrocarbons, it can be extended to treat nonplanar geometries by incorporating torsional effects into the electronic hopping term.^9,44,46–49^ In particular, the hopping integral *t*_μν_ between the InveST bridge and the radical moieties is modeled to vary as a cosine function of the torsional angle θ

2 $$t_{\mu \nu }\to t_{\mu \nu }\left(\theta \right)=t_{\mu \nu }\;\cos\theta$$

where both dihedral angles (on either side of the bridge) are rotated by the same amount, regardless whether in the same or opposite directions. The hopping reaches its maximum value when the molecule adopts a fully planar conformation, i.e., at θ = 0° or 180°. Torsional strain is introduced through a steric potential modeled as a squared sine function, *V*_steric_(θ) = sin^2^θ, assumed to be identical in both the ground and excited electronic states. All bond lengths are fixed to 1.4 Å, and all bond angles are fixed to 120°. This idealized π-skeleton follows the standard PPP parametrization and allows us to focus exclusively on the electronic effects of conjugation and torsion. Details on the model parameters and a quantitative comparison with the DFT-optimized ab initio geometries are provided in SI Section S1.2. Finally, the PPP Hamiltonian is solved by exact diagonalization, using the RASCI framework we introduced in ref 36 and described in detail in SI Section S1.3.

We start our discussion with the electronic structure of the 5AP-bridged diradical system (Figure 2a) at the PPP level, comparing it with high-level multireference ab initio CASSCF/QD-NEVPT2 calculations. This ab initio protocol has previously been successfully applied to molecular multiple-spin systems, where it was shown to provide robust and reliable excited-state exchange couplings.^50^ In the present case, the calculations employ an active space of 4 electrons in 4 orbitals (see SI Section S2.1). The resulting CASSCF molecular orbitals (MOs) in panel b show a doubly degenerate SOMO, each localized on one of the two radical units and labeled as SOMO_1_ and SOMO_2_ throughout the text. The HOMO and LUMO are centered on the InveST bridge. This orbital pattern is preserved over the range of torsional angles θ explored in this work. Results using a larger active space (6 electrons in 6 orbitals) are shown in SI Section S2.2. The corresponding PPP-RASCI calculations employ a RAS2 active space containing 4 electrons in 6 orbitals, accounting for the HOMO, SOMO_1_, SOMO_2_, and LUMO, along with two additional virtual orbitals mainly localized on the 5AP bridge (see SI Section S1.3). This RAS2 space provides a better quantitative agreement with the ab initio CASSCF/QD-NEVPT2 results, while preserving the same qualitative trends obtained with the smaller (4,4) RAS2 space (see SI Section S1.4).

**Figure 2:**
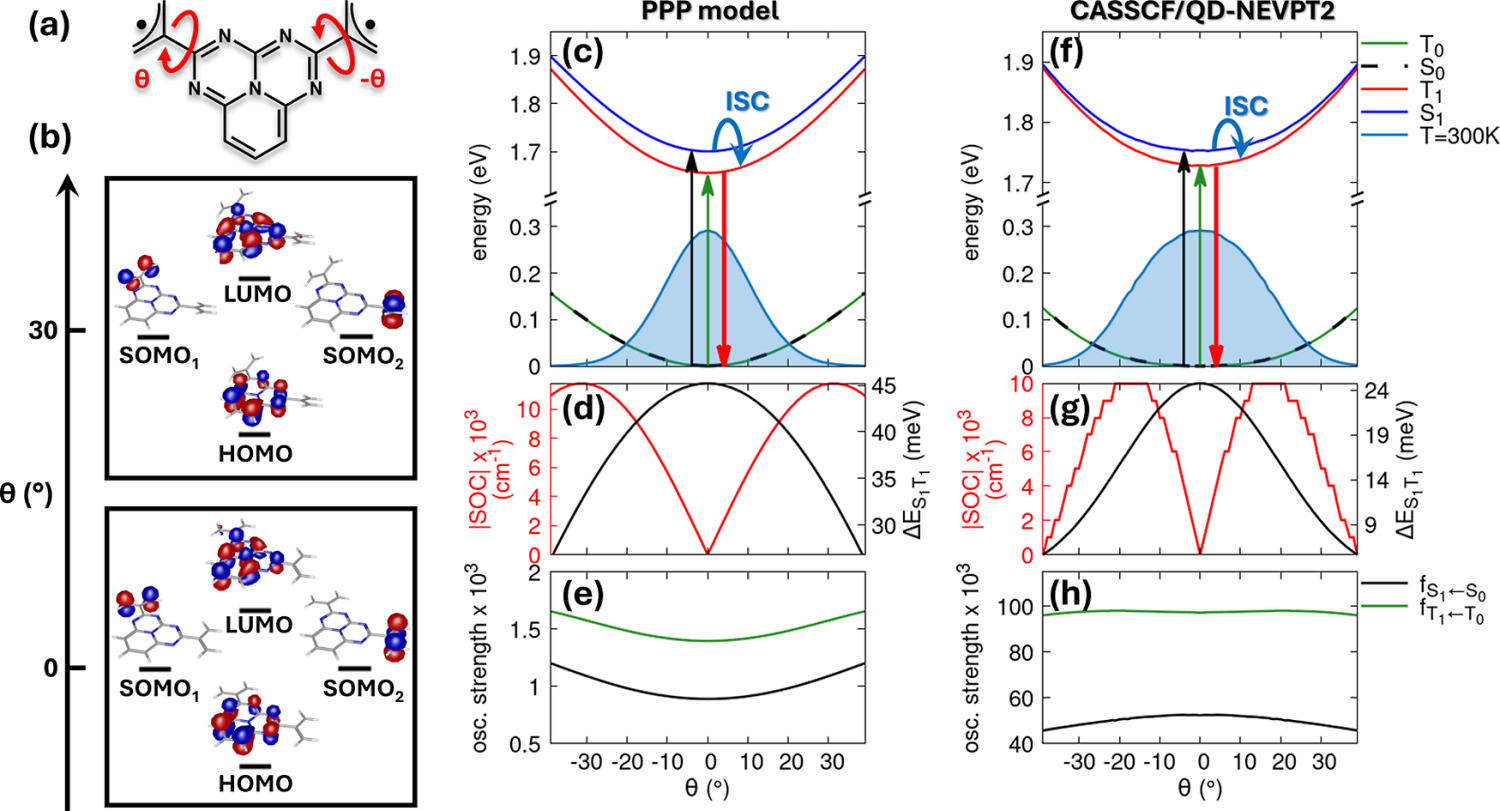
Electronic structure and photophysical properties of the 5AP-(allyl^•^)_2_. (a) Molecular structure with torsional coordinate θ around the bridge–radical connecting bonds. (b) CASSCF­(4,4) frontier MOs at θ = 0° and 30°. (c) PPP potential energy curves for S_0_, T_0_, S_1_, and T_1_ states and ground-state Boltzmann distribution at room temperature. (d) Singlet–triplet energy gap (black) and spin–orbit coupling magnitude (red) vs θ. (e) Oscillator strengths for S_0_ → S_1_ (black) and T_0_ → T_1_ (green) transitions. (f–h) CASSCF­(4,4)/QD-NEVPT2 results corresponding to panels (c–e). PPP calculations used the RASCI­(h,p,hp) approach with a (4,6) RAS2 space (see SI Section S1.3). PPP model parameters: *t* = −2.4 eV, ε_C_ = 0, *U*_C_ = 11.26 eV, ε_N_^aza^ = −3.5 eV, *U*_N_^aza^ = 15.5 eV, ε_N_^py^ = −13 eV, *U*_N_^py^ = 15 eV.

The isolated 5AP chromophore displays an experimental S_1_ energy of 1.95–2.00 eV, with the T_1_ state lying about 0.047 eV higher in energy.^33,34^ In the 5AP-(allyl^•^)_2_ diradical, the PPP-RASCI potential energy curves as a function of the torsional angle θ (panel c) show that both the ground and excited-state manifolds adopt a planar equilibrium geometry (θ_eq_ = 0°). However, they exhibit markedly different spin behavior. The singlet and triplet ground states (S_0_ and T_0_) remain degenerate across the entire torsional range, consistent with the expected absence of spin–spin interactions in the ground state. Upon photoexcitation, the population of the InveST LUMO enables interaction between the radicals, effectively switching on an exchange interaction in the excited state. This activation of inter-radical communication opens an energy gap between the first excited triplet (T_1_) and singlet (S_1_) states, with T_1_ stabilized below S_1_ over the range of θ values thermally accessible at room temperature (see the Boltzmann distribution calculated for the ground state in panel c). The magnitude of this ST splitting serves as a direct indicator of the light-induced radical–radical interaction, reaching a maximum at θ = 0° (panel (d), black curve). At θ = 0°, the PPP-RASCI calculations yield vertical transition energies of 1.655 eV (T_1_) and 1.700 eV (S_1_). Throughout the entire torsional coordinate, the S_1_ and T_1_ states retain their diradical character, with the two unpaired electrons remaining spatially separated on distinct radical units, rather than forming an intramolecular SOMO-to-SOMO charge-transfer state (see SI Section S1).

These PPP predictions are in good agreement with high-level CASSCF/QD-NEVPT2 calculations (panel f), which reproduce the same qualitative behavior, namely, degenerate singlet and triplet ground states and a positive, albeit slightly smaller, ST energy gap in the excited state (panel g, black curve), predicting transition energies of 1.728 eV (T_1_) and 1.753 eV (S_1_).

The ODMR mechanism requires spin-selective ISC, which in turn depends on the coupling between the singlet and triplet excited-state manifolds. This mixing is governed by SOC. As outlined in Section 1, SOC in InveST-bridged diradicals is enabled by thermal torsional fluctuations around the bonds linking the InveST core to the radical units. These out-of-plane distortions disrupt the conjugated π system planarity, triggering SOC. Notably, SOC is highly localized at the connection points between the bridge and radicals, due to the sharp 1/*r* ^3^ dependence of SOC on interatomic separation. As a result, the primary SOC contributions arise from the immediate vicinity of the InveST–radical linkages, where structural flexibility is the most significant. Within the PPP framework, the SOC operator takes the form:^9,51–54^

$$H_{SOC}=A\underset{\mu \nu }{\sum }\left(a_{\mu ↑}^{†}a_{\nu ↓}-a_{\nu ↑}^{†}a_{\mu ↓}\right)$$ 3

where *A* = −*i*(3.94 × 10^−4^) sin θ is the purely imaginary SOC matrix element in eV between neighboring carbon atoms 2p_*z*_ orbitals. Accordingly, in the PPP model, SOC is introduced as an additional spin-flipping hopping term with a purely imaginary amplitude *A* between atomic sites μ and ν, where these sites correspond to the junctions between the InveST bridge and the two radical units. The magnitude of *A* depends on the local torsional angle, which captures the conformational sensitivity of SOC activation. Figure 2d (red curve) shows the absolute value of the SOC matrix element between the S_1_ state and the *M*_S_ = ±1 sublevels of the T_1_ state, as calculated at the PPP level. No coupling is found between S_1_ and the *M*_S_ = 0 component of T_1_, in agreement with spin selection rules.^53^ As expected, the SOC vanishes exactly at θ = 0°, where the system is fully planar. However, even slight deviations from planarity lead to nonzero SOC values, suggesting that minimal torsional distortions may be sufficient to activate the ISC. The quantitative impact on ISC rates is discussed in Section 2.2. The SOC magnitude increases steadily with the torsional angle, reaching approximately 0.01 cm^−1^ at θ = ±30°. These values are in line with SOC strengths observed in other organic molecules known to undergo efficient ISC,^55,56^ indicating that thermally accessible twisting at the InveST–radical bonds can provide a pathway for spin-state transitions in this system. Calculations at the CASSCF/QD-NEVPT2 level (panel (g) red curve) show overall good agreement with the PPP results, yielding an SOC maximum of approximately 0.01 cm^−1^, identical in magnitude to that predicted by the PPP model. However, the positions of the maxima are shifted along the torsional coordinate: the ab initio SOC reaches its maximum at θ = ±18°. This shift reflects the inherently approximate nature of the PPP molecular orbitals involved in the spin-flipping hopping term, which only partially captures the torsional modulation of SOC in nonplanar geometries. Notably, a similar dependence of the SOC matrix element on the torsional angle was previously reported by Casanova et al. for the tetramethyleneethane molecule using the ab initio RAS-Spin-Flip theory level.^57^ In the present system, the S_1_–T_1_ SOC arises mainly from spin-flip excitations within each SOMO, namely, SOMO_1_^α^ → SOMO_1_^β^ and, equivalently, SOMO_2_^α^ → SOMO_2_^β^. The overall SOC magnitude thus reflects the combined contributions from these two localized spin-flip channels, whose relative phase and amplitude are modulated by the torsional angle θ at the junctions between the InveST bridge and radical fragments. In the CASSCF­(4,4)/QD-NEVPT2 calculations, the SOC reaches a minimum around θ ≃ 40°, where the local contributions from the two SOMOs interfere destructively, resulting in partial cancellation of the coupling.

The oscillator strengths for the S_0_ → S_1_ and T_0_ → T_1_ transitions, presented in panel e, show only minor variation as a function of the torsional angle θ. At the PPP level, these values are systematically underestimated by about 2 orders of magnitude compared to the CASSCF/QD-NEVPT2 results (panel h), a discrepancy that is well-known for this model and particularly pronounced for weak transitions such as those considered here.^58^ Nevertheless, the PPP calculations correctly reproduce the qualitative trend of the weak angular dependence. This limited sensitivity to torsion arises from the dominant localization of the S_1_ and T_1_ excited states on the InveST core, where the main optical transition occurs, with only minor involvement of the radical units. As a result, torsional fluctuations at the bridge–radical connections exert little influence on the transition probabilities.

The electronic structure of the 5AP-(allyl^•^)_2_ system supports a clear ODMR mechanism. Upon optical excitation, both S_1_ and T_1_ excited states are populated, with T_1_ lying energetically below S_1_. Thermal torsional fluctuations activate SOC between these states, thereby opening channels from S_1_ to specific T_1_ sublevels (*M*_S_ = ±1). This process can lead to a nonuniform population of triplet sublevels. As the excited states relax back to the degenerate S_0_/T_0_ ground-state manifold via radiative or nonradiative pathways, the sublevel-selective population is retained, resulting in ground-state triplet spin polarization.

The C_2_N_2_-bridged diradical system shown in Figure 3a presents electronic and photophysical properties closely resembling those of 5AP-(allyl^•^)_2_. As shown in panel b, the CASSCF­(4,4) frontier MOs retain the same characteristic pattern: the HOMO and LUMO are localized on the InveST bridge, while the doubly degenerate SOMO remains confined on the radical units. For the parent 1,3-diazete chromophore, no experimental data are available for the S_1_ and T_1_ states; however, high-level CC3/6-31+G­(d) calculations reported previously^35^ for the DFT-optimized geometry place S_1_ and T_1_ at 2.849 and 2.934 eV, respectively, indicating an inverted singlet–triplet gap. PPP-RASCI calculations for the C_2_N_2_-(trityl^•^)_2_ diradical again predict degenerate S_0_ and T_0_ states throughout the full range of torsional angles, along with a finite ST gap between S_1_ and T_1_, with T_1_ stabilized below S_1_ (panel c). At θ = 0°, the PPP-RASCI method yields transition energies of 1.437 eV (T_1_) and 1.479 eV (S_1_). These PPP potential energy curves show good overall agreement with the ab initio CASSCF­(4,4)/QD-NEVPT2 results (panel f), which reproduce the same qualitative behavior and predict transition energies of 1.899 eV (T_1_) and 1.953 eV (S_1_). In this case, the PPP-RASCI calculations using a (4,4) RAS2 space provide a good match with the ab initio CASSCF­(4,4)/QD-NEVPT2 results. Additional calculations with an enlarged (4,5) RAS2 space, including one additional virtual orbital, yield similar outcomes and are reported in SI Section S1.4. The torsional dependence and magnitude of the ST energy gap are well-reproduced (see the black curve in panels d and g), although the vertical excitation energies are slightly underestimated at the PPP level by ∼0.45 eV compared to QD-NEVPT2.

**Figure 3:**
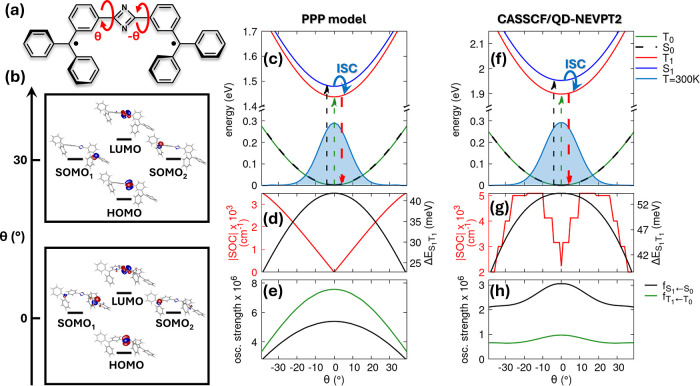
Electronic structure and photophysical properties of C_2_N_2_-(trityl^•^)_2_. (a) Molecular structure showing the trityl-based C_2_N_2_-diradical system with torsional angle θ around the bridge–radical bonds. (b) CASSCF­(4,4) frontier molecular orbitals at planar (θ = 0°) and twisted (θ = 30°) geometries. (c) PPP potential energy surfaces and ground-state thermal distribution at room temperature. (d) Torsional dependence of the S_1_–T_1_ energy gap (black) and spin–orbit coupling strength (red). (e) Oscillator strengths for S_0_ → S_1_ (black) and T_0_ → T_1_ (green). (f–h) CASSCF­(4,4)/QD-NEVPT2 relevant results. PPP calculations used the RASCI­(h,p,hp) approach with a (4,4) RAS2 space (see SI Section S1.3). PPP model parameters: *t* = −2.4 eV, ε_C_ = 0, *U*_C_ = 11.26 eV, ε_N_^aza^ = −3.5 eV, *U*_N_^aza^ = 12.34 eV, ε_N_^py^ = −13 eV, *U*_N_^py^ = 15 eV.

Differences arise in the SOC behavior. The absolute value of the SOC between S_1_ and T_1_ is smaller in this system compared to that of 5AP-(allyl^•^)_2_. While the PPP model predicts vanishing SOC at the planar geometry (θ = 0°, red curve in panel d), CASSCF­(4,4)/QD-NEVPT2 calculations show a small but finite SOC at equilibrium (red curve in panel g). This residual coupling results from a slight deviation from perfect planarity in the DFT-optimized geometry, specifically a dihedral angle of ∼0.5° between the InveST bridge and each trityl unit. The ab initio SOC reaches its maximum around θ = 10°–20° and shows a minimum near θ = ±39°, in contrast to the PPP SOC which places the maximum at θ = ±39°. Despite these differences in angular dependence, both methods yield SOC values of a comparable magnitude. This discrepancy originates from the inherently approximate nature of the PPP molecular orbitals involved in the spin-flipping hopping term and from the fact that the PPP model is primarily designed for planar π-conjugated systems. As a result, the PPP framework cannot fully describe the more complex orbital mixing and correlation effects that govern SOC in nonplanar architectures such as C_2_N_2_-(trityl^•^)_2_.

A key feature of the C_2_N_2_-(trityl^•^)_2_ system lies in its optical behavior: the lowest excited states (S_1_ and T_1_) are optically dark due to the symmetric character of the C_2_N_2_ bridge (panels e and h). As a result, photoexcitation must proceed through higher-lying excited states, which subsequently relax via internal conversion to the S_1_ and T_1_ manifolds (see SI Section S4). SOC between S_1_ and T_1_ then enables ISC from S_1_ to specific T_1_ sublevels (*M*_S_ = ±1), followed by nonradiative internal conversion (dashed red arrow in panels c and f) that can lead to triplet spin polarization in the ground state.

Turning to the 5AP-(trityl^•^)_2_ diradical shown in Figure 1d, the system retains the characteristic degeneracy of S_0_ and T_0_ observed in the previously discussed cases (see SI Section S5). However, although an energy gap still opens between S_1_ and T_1_, with T_1_ lying below S_1_, the magnitude of this gap is substantially reduced compared to the two smaller diradical analogs. At θ = 0°, the S_1_–T_1_ splitting reaches only 17 meV at the PPP-RASCI­(h,p,hp) level with a (4,4) RAS2 space and 5 meV in CASSCF­(4,4)/QD-NEVPT2 calculations. This already small gap decreases further at nonzero torsional angles. The SOC properties are similarly weakened: the maximum absolute value of the SOC matrix element between S_1_ and the *M*_S_ = ±1 sublevels of T_1_ is 4.5 × 10^−4^ cm^−1^ at the PPP level and 10^−3^ cm^−1^ at the CASSCF­(4,4)/QD-NEVPT2 level. This represents a reduction of nearly 2 orders of magnitude compared to the 5AP-(allyl^•^)_2_ system (see Figure 2d,g). The combination of a minimal S_1_–T_1_ energy gap and an extremely weak SOC makes 5AP-(trityl^•^)_2_ unsuitable for efficient ODMR operation.

To understand the design principles that govern radical–radical interactions in InveST-bridged systems, we examine the SOMO–LUMO exchange integral at the PPP level (see SI Section S1.5) for four diradical variants, including now also the C_2_N_2_-(allyl^•^)_2_ (Figure 4a). At the planar geometry (θ = 0°), the exchange integral follows the trend: C_2_N_2_-(allyl^•^)_2_ > 5AP-(allyl^•^)_2_ > C_2_N_2_-(trityl^•^)_2_ > 5AP-(trityl^•^)_2_. The larger value for C_2_N_2_-(allyl^•^)_2_ reflects its stronger SOMO–LUMO overlap, with an exchange integral about five times larger than that in the corresponding system with the 5AP bridge. This result highlights the direct connection between the strength of the exchange interaction and the degree of SOMO–LUMO overlap (see panel b). Among these systems, C_2_N_2_-(allyl^•^)_2_ and 5AP-(allyl^•^)_2_ show the most pronounced SOMO–LUMO overlap, leading to an efficient coupling between the radical centers. In contrast, 5AP-(trityl^•^)_2_ exhibits only a weak orbital interaction due to its extended framework, resulting in negligible excited-state radical–radical coupling, whereas C_2_N_2_-(trityl^•^)_2_ shows intermediate behavior with moderate overlap and exchange strength. The exchange integral calculated from CASSCF­(4,4) MOs using the Multiwfn package^59,60^ confirms the same trend and spans a comparable energy range (see panels c and d).

**Figure 4:**
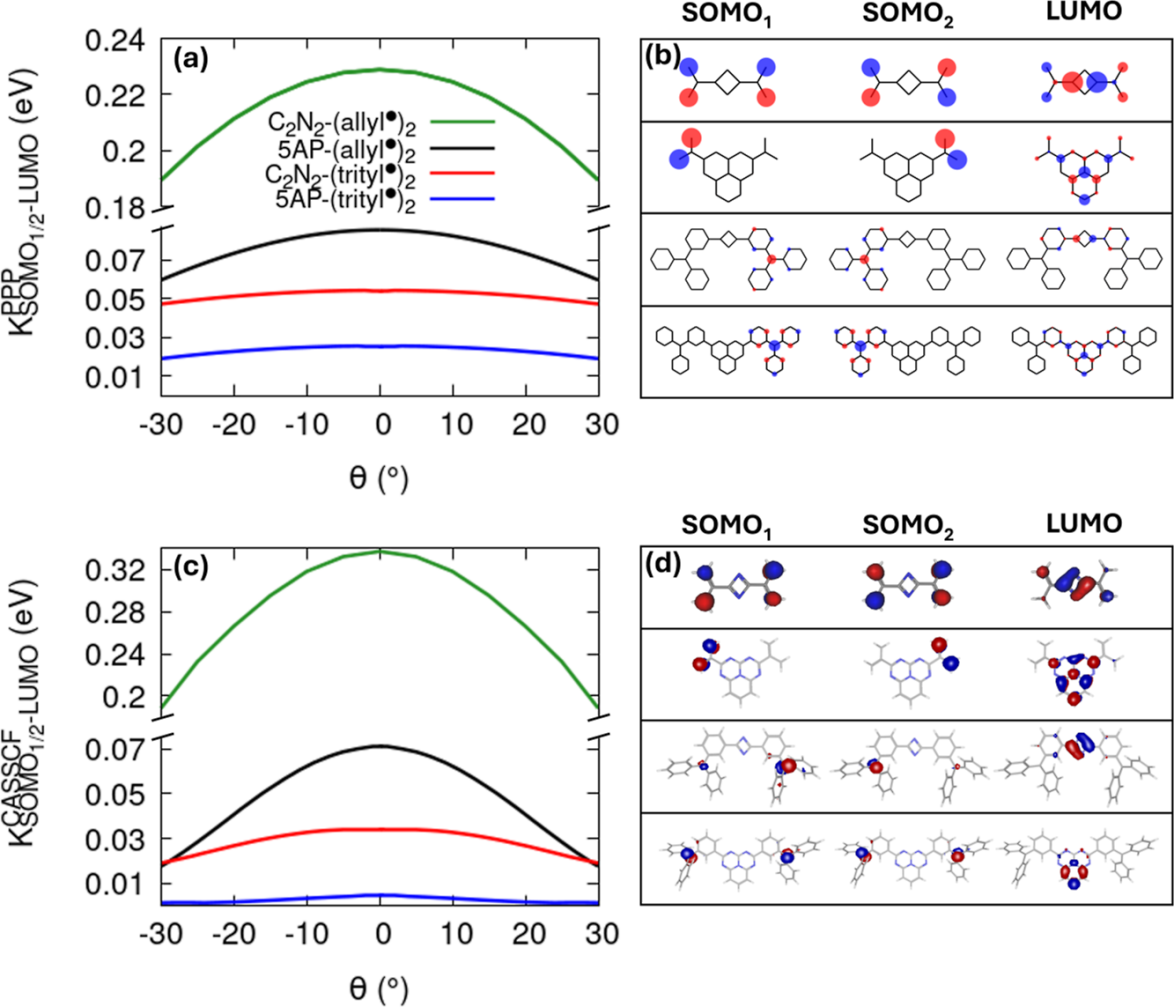
Exchange interactions in InveST-bridged diradicals. (a) SOMO–LUMO exchange integral as a function of torsional angle θ for C_2_N_2_-(allyl^•^)_2_ (green), 5AP-(allyl^•^)_2_ (black), C_2_N_2_-(trityl^•^)_2_ (red), and 5AP-(trityl^•^)_2_ (blue) at the PPP Hartree–Fock level. (b) PPP frontier MOs sketches (SOMO_1_, SOMO_2_, and LUMO) for the three systems at θ = 0°. (c,d) Relevant results obtained with CASSCF MOs (active space (4,4)). PPP model parameters for C_2_N_2_-(allyl^•^)_2_: *t* = −2.4 eV, ε_C_ = 0, *U*_C_ = 11.26 eV, ε_N_^aza^ = −4 eV, *U*_N_^aza^ = 12.34 eV. Model parameters for 5AP-(trityl^•^)_2_: *t* = −2.4 eV, ε_C_ = 0, *U*_C_ = 11.26 eV, ε_N_^aza^ = −4 eV, *U*_N_^aza^ = 15.5 eV, ε_N_^py^ = −13 eV, *U*_N_^py^ = 15 eV. Model parameters for 5AP-(allyl^•^)_2_ in Figure 2 and for C_2_N_2_-(trityl^•^)_2_ in Figure 3.

### 2.2 The ODMR Mechanism for InveST-Bridged Diradicals

The ODMR mechanism in InveST-bridged diradicals emerges from a sequence of spin-dependent photophysical processes that originate in the thermally populated conformational ensemble of the ground state. At room temperature, the degenerate S_0_–T_0_ ground state spans a distribution of torsional geometries, with an appreciable population of twisted conformations around the bonds connecting the InveST core to the radical units. This conformational flexibility plays a key role in activating the SOC pathways that drive the subsequent photophysical dynamics. Upon photoexcitation, the behavior of the system depends on the specific diradical structure and the oscillator strengths of its low-lying electronic transitions. In the case of 5AP-(allyl^•^)_2_, direct excitation can populate the S_1_ and T_1_ states. By contrast, C_2_N_2_-(trityl^•^)_2_ exhibits vanishing oscillator strengths for both S_0_ → S_1_ and T_0_ → T_1_ transitions, necessitating excitation to higher-lying states. Nonetheless, Kasha’s rule ensures rapid, spin-preserving internal conversion from these higher excited states down to S_1_ and T_1_, effectively funneling excitation into the same manifold regardless of the initial excitation pathway.

Once the S_1_ and T_1_ states are populated, they serve as key players in the ODMR mechanism. Thermal fluctuations in the torsional angles dynamically modulate the SOC strength, creating pathways for ISC between S_1_ and the *M*_S_ = ±1 sublevels of T_1_. The efficiency of ISC depends on two key factors: (i) the magnitude of SOC, which depends on the instantaneous molecular conformation, and (ii) the S_1_–T_1_ energy gap, with ISC rates decaying exponentially as this gap widens. This mechanism is most effective when S_1_ and T_1_ are nearly degenerate, as small energy gaps enhance the probability of ISC. However, this same condition also increases the likelihood of reverse intersystem crossing (RISC), especially at room temperature. The interplay between ISC and RISC determines the distribution of the excited-state population. When ISC dominates over RISC, the singlet population is preferentially shelved in the triplet state, leading to spin polarization within the excited-state manifold.

The final step in achieving ground-state spin polarization relies on preserving the spin-selective character of the decay pathways from the excited states. Whether relaxation occurs via radiative emission or internal conversion, the transition back to the S_0_/T_0_ manifold must preserve the spin polarization established in the excited state. Critically, the absence of SOC between S_0_ and T_0_—a consequence of the nodal structure of the HOMO and SOMO in InveST-bridged diradicals—prevents spin mixing during de-excitation. As a result, the spin polarization generated in the excited state can be efficiently transferred and maintained in the ground-state population.

To validate this mechanism, we must calculate the rates for ISC, RISC, and emission as functions of the molecular conformation. This begins with constructing a diabatic model by diabatizing the potential energy curves associated with the S_0_, T_0_, S_1_, and T_1_. The model includes four diabatic states: two singlets, corresponding to a neutral state |^1^N⟩ and a multiresonant charge-transfer state |^1^MRCT⟩, and two corresponding triplets, |^3^N⟩ and |^3^MRCT⟩. The neutral states |^1^N⟩ and |^3^N⟩ define the reference energy (set to zero). The diabatic energy of |^1^MRCT⟩ is set to 2*z* and that of |^3^MRCT⟩ is set to 2*s*. The singlet states are coupled by torsion-dependent matrix element −τ­(θ), while the triplet coupling is given by −β­(θ). Following the El-Sayed rule, we set the SOC between |^1^N⟩ and |^3^MRCT⟩, and between |^3^N⟩ and |^1^MRCT⟩, both through a constant matrix element denoted as *V*_SOC_. Its value was determined by requiring that the matrix element |⟨S_1_|*V*_SOC_|T_1_⟩|, evaluated from the eigenstates of the diabatic Hamiltonian, reproduces the θ-dependent SOC profile obtained from PPP-RASCI or QD-NEVPT2 calculations. The diabatic Hamiltonian reads

4 $$H=\left(\begin{array}{llll} 0 & 0 & -\tau \left(\theta \right) & V_{SOC}\\ 0 & 0 & V_{SOC} & -\beta \left(\theta \right)\\ -\tau \left(\theta \right) & V_{SOC} & 2z & 0\\ V_{SOC} & -\beta \left(\theta \right) & 0 & 2s \end{array}\right)+\frac{\hbar \omega_{t}}{2}\left(\theta^{2}+p_{\theta }^{2}\right)+a\theta^{4}$$

where the basis states are ordered as {|^1^N⟩, |^3^N⟩, |^1^MRCT⟩, |^3^MRCT⟩} and ω_*t*_ is the frequency associated with the conformational coordinate, *p*_θ_ being the conjugate momentum. The torsional dependence of the off-diagonal couplings is modeled as −τ­(θ) = −τ_0_ cos­(2θ) sin­(2θ) for the singlet manifold and −β­(θ) = −β_0_ cos­(2θ) sin­(2θ) for the triplet manifold. A quartic (anharmonic) restoring potential is employed to mimic the potential energy curves of the ground and excited states vs θ. Additional details can be found in SI Section S6.5.

ISC and RISC processes are driven by tiny SOC interactions that can be treated perturbatively. To compute the relevant transition rates, we first diagonalize the Hamiltonian in eq 3 with the *V*_SOC_ value set to zero. Under this approximation, the singlet and triplet manifolds become decoupled, allowing them to be treated separately (see SI Section S5.2). The resulting vibronic energy levels in the T_1_ and S_1_ manifolds are shown as gray and blue lines, respectively, in Figure 5a,c. Since internal conversion is extremely rapid (typically on the order of tens of femtoseconds), we assume that ISC occurs from a thermally equilibrated population of S_1_ vibronic states. This thermal distribution is shown by the blue shaded area in Figure 5a–c. Transition rates between singlet and triplet states are calculated using the Fermi Golden Rule $k_{ISC}^{i\to j}=|\left\langle i\left|V_{SOC}\right|j\right\rangle {|}^{2}S_{ij}2\pi /\hbar$ where *S*_*ij*_ measures the overlap between states |*i*⟩ and |*j*⟩. Each state is modeled with a Gaussian line shape, with width σ related to the inverse of the relaxation time τ as $\sigma ={\left(2\pi \tau \sqrt{2\;\log2}\right)}^{-1}$ . RISC rates are obtained from the corresponding ISC rates using a detailed balance. Panels b and d report ISC and RISC rates for four different τ values. The chosen range (30–60 fs) corresponds to realistic lifetimes of vibronic states in organic molecules.^61^ For the 5AP-(allyl^•^)_2_ system, ISC rates are on the order of 1 × 10^5^ s^−1^, while RISC rates reach 1 × 10^4^ s^−1^, with minimal dependence on the τ value because of the small S_1_–T_1_ gap. In contrast, the C_2_N_2_-(trityl^•^)_2_ system exhibits lower rates, down to 10^3^ s^−1^ for both ISC and RISC, due to its smaller SOC and the larger S_1_–T_1_ energy gap with respect to the 5AP-(allyl^•^)_2_. For the C_2_N_2_-(trityl^•^)_2_ system, the calculated ISC and RISC rates tend to converge as relaxation time τ increases. In particular, at τ = 60 fs, the two processes exhibit nearly identical rate constants. This behavior arises from the combined effect of the narrower Gaussian line shape (i.e., smaller σ) associated with each vibronic state at longer τ and the larger energy gap between the S_1_ and T_1_ states compared to 5AP-(allyl^•^)_2_, thus reducing overlapping vibronic levels, leading to comparable ISC and RISC efficiencies.

**Figure 5:**
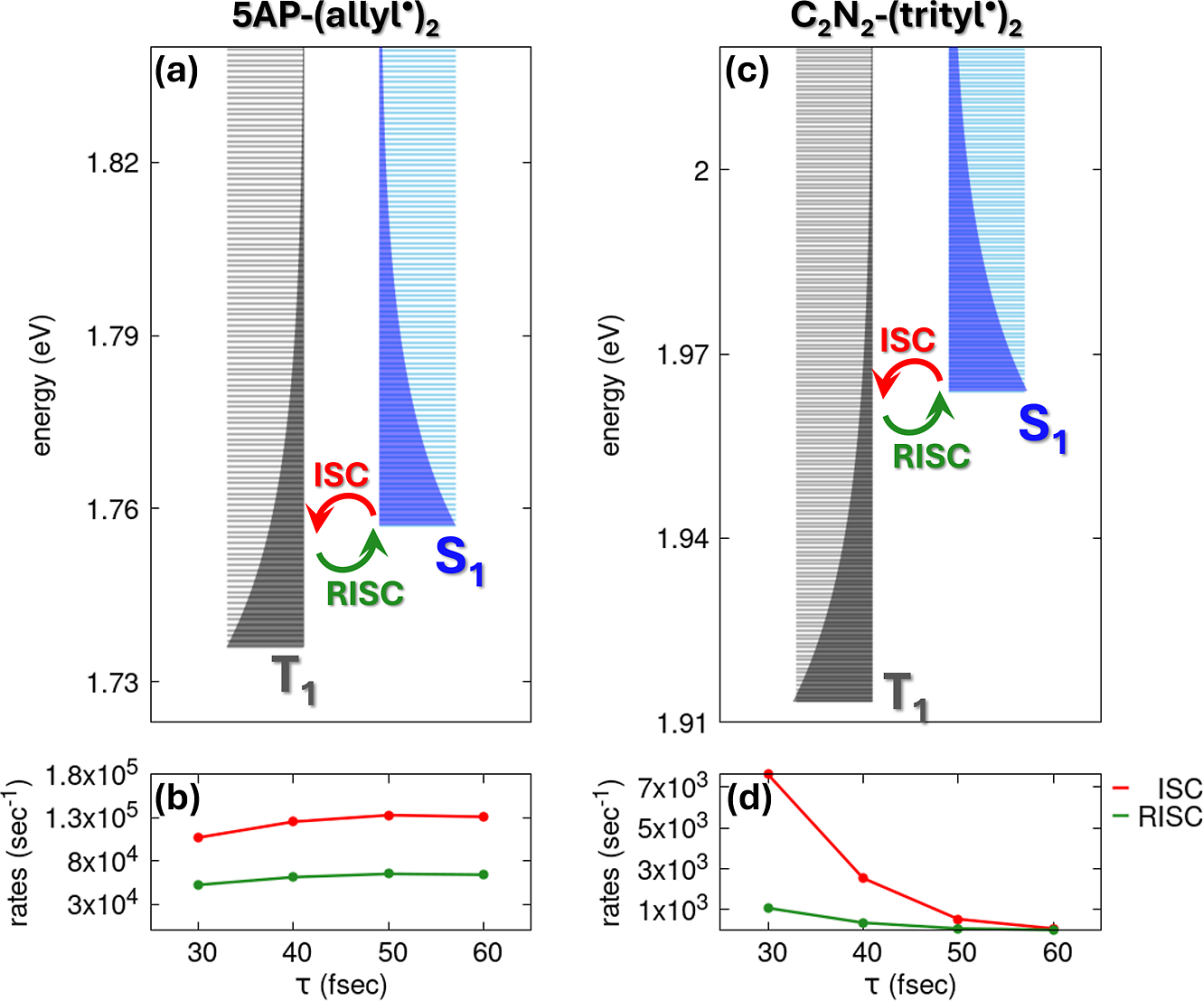
A schematic representation of the vibronic calculation of ISC rates and RISC rates for 5AP-(allyl^•^)_2_ (panel a) and C_2_N_2_-(trityl^•^)_2_ (panel c) starting from the CASSCF­(4,4)/QD-NEVPT2 results shown in Figures 2 and 3. In both panels, gray and blue lines show the energy of the vibronic triplet and singlet eigenstates, respectively. The global ISC rate is calculated summing all the rates of the S_1_ to T_1_ processes, averaging on the thermal population of singlet states (graphically represented by the blue shaded area). RISC rates are evaluated from the ISC rate by imposing the microscopic reversibility condition. ISC and RISC rates calculated for different values of the relaxation time τ are reported in panels b and d. Parameters for 5AP-(allyl^•^)_2_: τ_0_ = 0.17 eV, β_0_ = 0.24 eV, 2*z* = 1.76 eV, 2*s* = 1.74 eV, ℏω_*t*_ = 8.7 × 10^−4^ eV, *a* = −0.15 eV, *V*_SOC_ = −0.09 eV. Parameters for C_2_N_2_-(trityl^•^)_2_: τ_0_ = 0.21 eV, β_0_ = 0.22 eV, 2*z* = 1.96 eV, 2*s* = 1.91 eV, ℏω_*t*_ = 6.2 × 10^−4^ eV, *a* = −0.08 eV, *V*_SOC_ = −0.05 eV.

The radiative rate is calculated as *k*_*fi*_ = (ω_*fi*_^3^μ_*fi*_^2^)/(3πε_0_ℏc^3^) where ω_*fi*_ is the transition frequency and μ_*fi*_ is the transition dipole moment between the initial and final states.^62^ This rate is calculated as a function of the conformational coordinate θ, yielding a θ-dependent emission rate. We then perform a thermal average over this distribution, weighting each contribution by the energy of the corresponding fluorescent state at that θ value. For 5AP-(allyl^•^)_2_, the resulting thermally averaged radiative rate is 3.5 × 10^7^ s^−1^, whereas for the C_2_N_2_-(trityl^•^)_2_, it is essentially zero, as already observed when discussing the calculated oscillator strength as a function of θ.

In summary, among the systems investigated, 5AP-(allyl^•^)_2_ emerges as the most promising candidate for realizing optically addressable spin states. This molecule combines a sizable radiative decay rate, a finite ISC rate, and comparatively slower RISC, thus satisfying the key requirements for effective spin polarization via an ODMR mechanism. Additional ISC and RISC rates computed from the diabatized PPP results are provided in the SI (Figure S7). While the rates for the C_2_N_2_-bridged diradical align with those obtained from the ab initio-based model, the ISC rates for 5AP-(allyl^•^)_2_ are slightly smaller due to the larger S_1_–T_1_ gap predicted at the PPP level. This highlights the critical role of carefully tuning both the excited-state singlet–triplet energy gap and the SOC strength to engineer optimal optical–spin interfaces in organic diradicals.

## 3 Conclusions

In conclusion, we have introduced a molecular design strategy for achieving optically addressable spin states in organic diradicals bridged by InveST units, molecular dyes with an inverted ST energy gap. Using a combination of the PPP model and high-level multireference ab initio methods, we showed that these systems can function as tunable optical–spin interfaces capable of supporting ISC, spin polarization, and potentially ODMR activity. A key feature of these diradicals is the absence of exchange interaction between the radical centers in the ground state, resulting in degenerate singlet and triplet ground states. Upon optical excitation, either directly into the low-lying excited states or via internal conversion from higher-lying states, the InveST LUMO becomes populated and mediates exchange coupling between the radicals, opening a finite energy gap between the S_1_ and T_1_ states. This photoinduced switching of radical–radical interaction is central to enabling spin dynamics. Torsional fluctuations around the bonds connecting the InveST core to the radical units are thermally populated at room temperature, and these distortions break molecular planarity, thereby activating SOC. The resulting SOC selectively couples S_1_ to the *M*_S_ = ±1 sublevels of T_1_, opening a pathway for spin-selective ISC. Estimated ISC, RISC, and radiative decay rates indicate that 5AP-(allyl^•^)_2_ exhibits the most favorable balance of strong SOC, efficient ISC, and suitable photophysical properties for ODMR operation. In contrast, C_2_N_2_-(trityl^•^)_2_ and 5AP-(trityl^•^)_2_ show a reduced SOC and weaker excited-state exchange interactions, limiting their ODMR potential. For completeness, we also evaluated the lowest quintet states at the PPP-RASCI level, finding them higher in energy (1.79 eV for 5AP-(allyl^•^)_2_, 1.61 eV for C_2_N_2_-(trityl^•^)_2_, and 2.00 eV for 5AP-(trityl^•^)_2_) than T_1_ and S_1_ levels, confirming that the photophysics is governed by singlet and triplet excitations, with negligible quintet contribution.

Altogether, these findings position InveST-bridged diradicals as a compelling molecular platform for quantum technologies. Their unique combination of ground-state spin degeneracy, light-induced exchange interactions, and SOC pathways establishes a chemically tunable route to spin control at the molecular level. Importantly, the modular architecture of these systems offers large opportunities for rational optimization: by fine-tuning the electronic structure of the InveST bridge and tailoring the nature of the radical units, both the S_1_–T_1_ energy gap and the SOC strength can be modulated. The promising results obtained for 5AP-(allyl^•^)_2_ should be viewed as a proof of concept for this design strategy. While simple allylic radicals are not synthetically stable, heteroatom analogues such as nitronyl nitroxide and verdazyl radicals, or stable allyl surrogates like BDPA (Koelsch) radicals, offer experimentally accessible alternatives with similar electronic characteristics.^63^ These well-established open-shell systems provide viable routes to implementing InveST-based diradicals with enhanced ISC efficiency and robust spin polarization, key ingredients for realizing scalable and flexible molecular platforms for optically addressable spin qubits.

## Supplementary Material



## Data Availability

The data that support the findings of this study are available from the corresponding author upon reasonable request.

## Notes

Section S1: PPP model technicalities; Section S2: additional CASSCF/QD-NEVPT2 results obtained for different active spaces; Section S3: electronic character of the S_1_ and T_1_ excited states; Section S4: results on higher-lying excited states in C_2_N_2_-(trityl^•^)_2_; Section S5: results on the torsional angle dependence in 5AP-(trityl^•^)_2_; Section S6: details on the ISC and RISC rate constants calculations; and Section S7: Cartesian coordinates for the DFT-optimized geometries (PDF)
