# Supplementary material for: Organic Diradicals Bridged by Inverted Singlet–Triplet Units for Optical–Spin Interfaces
Source: J Chem Theory Comput. 2025 Nov 29;22(3):1465–75. doi: 10.1021/acs.jctc.5c01571 (PMC12895423; doi:10.1021/acs.jctc.5c01571)
Supplement: Supplementary file 1 [file ct5c01571_si_001.pdf]

Supplementary Information

Organic Diradicals Bridged by Inverted Singlet–Triplet Units for Optical–Spin Interfaces

Lorenzo Savi,[†,‡] Marco Tommaso Barreca,[†,‡] Matteo Bedogni,[†] and Francesco Di Maiolo*[,†]

†*Department of Chemistry, Life Science and Environmental Sustainability, Università di Parma, 43124 Parma, Italy.*

‡*Contributed equally to this work*

E-mail: francesco.dimaiolo@unipr.it

Table of Contents

S1 The Pariser-Parr-Pople Model Technicalities **S3**

S1.1 Modeling Electron Correlation in the PPP Hamiltonian S3

S1.2 The PPP Model Parameters S5

S1.3 The PPP-RASCI approach S6

S1.4 The RAS2 Space Dimension Dependence S8

S1.5 The PPP SOMO-LUMO exchange integral S9

S2 *Ab initio* Computational Details **S15**

S2.1 Computational Setup and Electronic Structure Methods S15

S2.2 Active Space Dependence: QD-NEVPT2 Energies and CASSCF Molecular Orbitals S16

S3 Electronic Character of S_1 and T_1 Excited States **S19**

S4 Higher-lying excited states in C_2N_2-(trityl$^\bullet$)$_2$ **S21**

S5 Torsional Angle Dependence in 5AP-(trityl$^\bullet$)$_2$ **S23**

S6 ISC and RISC Rate Calculation Details **S27**

S6.1 The Diabatization Procedure S27

S6.2 Quantum Treatment of the Torsional Degree of Freedom θ S27

S7 Cartesian coordinates **S31**

References **S47**

S1 The Pariser-Parr-Pople Model Technicalities

S1.1 Modeling Electron Correlation in the PPP Hamiltonian

In the real space formulation of the PPP Hamiltonian (Eq. 1, main text), the inter-site electron-electron repulsion term $V_{\mu\nu}$ is modeled using the Ohno expression:[1–3]

$$V_{\mu\nu} = \frac{e^2}{4\pi\varepsilon_0} \Bigg/ \sqrt{r_{\mu\nu}^2 + \left(\frac{\varepsilon_r e^2}{4\pi\varepsilon_0(U_\mu + U_\nu)}\right)^2} \tag{S1}$$

where the relative dielectric constant ε_r is set to 2 to represent a typical organic environment.[3] The PPP Hamiltonian is expressed in a real-space basis comprising all possible configurations of n π-electrons distributed across N atomic sites. Basis states are chosen as eigenstates of the z-component spin operator, S_z. To identify singlet and triplet states, the PPP Hamiltonian is diagonalized in both $S_z = 0$ and $S_z = 1$ subspaces: singlets appear only in the $S_z = 0$ subspace, while triplets are present in both. As the number of sites N increases, the size of the basis grows exponentially, resulting in extremely large and sparse Hamiltonian matrices. The lowest-lying eigenstates and eigenvalues are obtained using the implicitly restarted Lanczos algorithm, as implemented in the ARPACK library.[4]

To enable direct comparison with quantum chemical methods, the real-space PPP model can be rewritten in the molecular orbital (MO) basis. This is achieved by defining the operators $b_{k\sigma}^{(\dagger)} = \sum_\mu c_{\mu,k} a_{\mu\sigma}^{(\dagger)}$, which annihilate (create) an electron with spin σ in the k-th molecular orbital. Here, $c\mu k$ are the MO coefficients obtained from the diagonalization of the Fock operator:

$$F_{PPP} = \sum_\mu \left(\varepsilon_\mu + J_{\mu\mu} - K_{\mu\mu}\right) n_\mu + \sum_{\mu\nu,\mu\neq\nu} \left(-t_{\mu\nu} - K_{\mu\nu}\right) \sum_\sigma \left(a_{\mu\sigma}^\dagger a_{\nu\sigma} + a_{\nu\sigma}^\dagger a_{\mu\sigma}\right) \tag{S2}$$

The zero differential overlap (ZDO) approximation is employed, leading to a diagonal form

of the Coulomb operator:

$$J_{\mu\mu} = \sum_{\lambda=1}^{N} \left(P_{\lambda\lambda} - Z_{\lambda}\right) V_{\lambda\mu} \tag{S3}$$

while the exchange operator reads:

$$K_{\mu\nu} = \left(P_{\mu\nu}/2 - Z_{\nu}\delta_{\mu\nu}\right) V_{\mu\nu} \tag{S4}$$

In Eqs. S3 and S4 the density matrix elements are defined as :

$$P_{\mu\nu} = 2 \sum_{k=1}^{SOMO-2} c_{k\mu}c_{k\nu} + c_{SOMO_1,\mu}c_{SOMO_1,\nu} + c_{SOMO_2,\mu}c_{SOMO_2,\nu} \tag{S5}$$

where in the first term k runs on the doubly occupied MOs in the ground state configuration, while the second and third terms account for the doubly degenerate SOMO of the two radical units.

Once the HF MOs are obtained for the ground state configuration $|g\rangle$, excited configurations are constructed by transferring one (single), two (double), three (triple), or more electrons from occupied to virtual MOs. The PPP Hamiltonian is then written in this basis and diagonalized using the Configuration Interaction (CI) method.

The molecular excited states S_1 and T_1 obtained from the diagonalization of the PPP Hamiltonian, are used to calculate the oscillator strengths for the $S_0 \rightarrow S_1$ and $T_0 \rightarrow T_1$ transitions. The electric dipole moment operator reads:

$$\vec{\mu} = \mu_x\vec{i} + \mu_y\vec{j} = \sum_{\mu}(Z_{\mu} - n_{\mu})(x_{\mu}\vec{i} + y_{\mu}\vec{j}) \tag{S6}$$

where x_{μ}, y_{μ} are the Cartesian coordinates of site μ within the molecular plane. The oscillator strength for the $|f\rangle \leftarrow |g\rangle$ transition reads:[5]

$$f_{fg} \quad = \quad \frac{2}{3}\frac{m_e}{\hbar e^2}\omega_{fg}|\mu_{fg}|^2 \tag{S7}$$

where m_e is the electron mass, e the electron charge, $\omega_{fg} = E_f - E_g$ is the relevant transition energy, and $\mu_{fg} = \langle f|\mu|g\rangle$ is the transition dipole moment.

S1.2 The PPP Model Parameters

Carbon atom PPP parameters are well-established and widely transferable across different classes of π-conjugated systems.[2,3,6–8] In this work, we adopt a standard parametrization with a carbon on-site energy set to zero, a Hubbard U_C of 11.26 eV, and a nearest-neighbor C-C hopping integral of $t = -2.4$ eV. In contrast, parametrizing nitrogen atoms remains less standardized, as no universally accepted set of PPP values exists for them.[2,3,7,9–18] Best agreement with QD-NEVPT2 results for 5AP-(allyl$^\bullet$)$_2$ diradicals is achieved by using Hubbard U values of 15 eV for pyrrolic nitrogen and 15.5 eV for aza nitrogen, as proposed in Ref. 19. The on-site energy for pyrrole nitrogen is set to –13 eV, while aza nitrogen site energies are taken as –3.5 eV in 5AP-(allyl$^\bullet$)$_2$ and –4 eV in 5AP-(trityl$^\bullet$)$_2$. For the C_2N_2-bridged diradical, which features two aza nitrogens and no pyrrolic nitrogen atoms, the parametrization was based on the PPP values for aza nitrogens in porphines reported in Ref. 18. Specifically, the Hubbard U was taken directly from that work (12.34 eV), while the site energy was slightly adjusted to –3.5 eV to improve agreement with high-level calculations. Hopping integrals for C–N bonds were set equal to the C–C values, consistent with earlier studies.[19–21] For computational simplicity, the molecular geometry was simplified by setting all bond angles to 120° and all bond lengths to 1.4 Å. For the trityl radical units, DFT-optimized geometries (Tabs. S10 and S13) showed a propeller-like structure, with dihedral angles of approximately 32° between the central carbon and each of the three phenyl rings. As a result, the hopping integrals between the central carbon and its phenyl substituents were scaled by $\cos(32°)$. Finally, as discussed in the main text, the torsional degrees of freedom around the InveST–radical connecting bonds modulate the electronic coupling between fragments. The hopping integral $t_{\mu\nu}$ connecting the radical sites to the InveST core was modeled with a cosine dependence on the torsional angle θ, with the two

dihedrals rotated by the same amount, regardless of whether the twisting occurs in the same or opposite directions. Table S1 summarizes the deviations in heavy-atom bond lengths and angles between the DFT-optimized and PPP-idealized structures. In all cases, bond length deviations are modest, typically within 0.04-0.05 Å on average, with maxima below 0.09 Å, and bond angle deviations are limited to a few degrees, with RMS values of $\sim$1-2°. The equilibrium torsional angle remains $\theta = 0°$ for all three diradicals.

Table S1: Deviations between DFT-optimized heavy-atom geometries and the idealized PPP geometries (all bonds 1.40 Å, all angles 120°; hydrogens omitted). For the C_2N_2 bridge in C_2N_2-(trityl$^\bullet$)$_2$, the PPP model uses 90° angles at the central C-N-C/N-C-N junction (120° elsewhere). In all cases the equilibrium torsional angle is planar ($\theta = 0°$).

System	Bond lengths vs 1.40 Å		Bond angles vs 120°	
	RMSD (Å)	Max $\lvert\Delta r\rvert$ (Å)	RMSD (°)	Max $\lvert\Delta\alpha\rvert$ (°)
5AP-(allyl$^\bullet$)$_2$	0.055	0.090	2.30	5.64
C_2N_2-(trityl$^\bullet$)$_2$	0.055	0.090	2.30	5.70
5AP-(trityl$^\bullet$)$_2$	0.037	0.082	1.64	5.61

S1.3 The PPP-RASCI approach

To make the PPP problem numerically tractable, we use the Restricted Active Space Configuration Interaction (RASCI) strategy to PPP, as developed in Ref. 20. Within this scheme,[22,23] the HF-MOs obtained from the PPP Hamiltonian are divided into three subspaces: RAS1, RAS2, and RAS3, ordered by increasing energy. RAS1 includes occupied orbitals, RAS2 comprises a mix of occupied and low-lying virtual MOs, and RAS3 is composed of higher-energy virtual orbitals. The simplest RASCI variant includes all possible configurations within RAS2, corresponding to a complete active space CI (CASCI). This can be extended through the hole-particle approximation, which allows a fixed number of excitations out of RAS1 (holes) and a fixed number of electrons (particles) in RAS3. This increases the correlation effects captured in the RASCI solution. For instance, the RASCI(h,p) scheme allows only one excitation either out of RAS1 into RAS2 or from RAS2 into RAS3,[24] while RASCI(h,p,hp) further includes single excitations from RAS1 into RAS3. The precision of

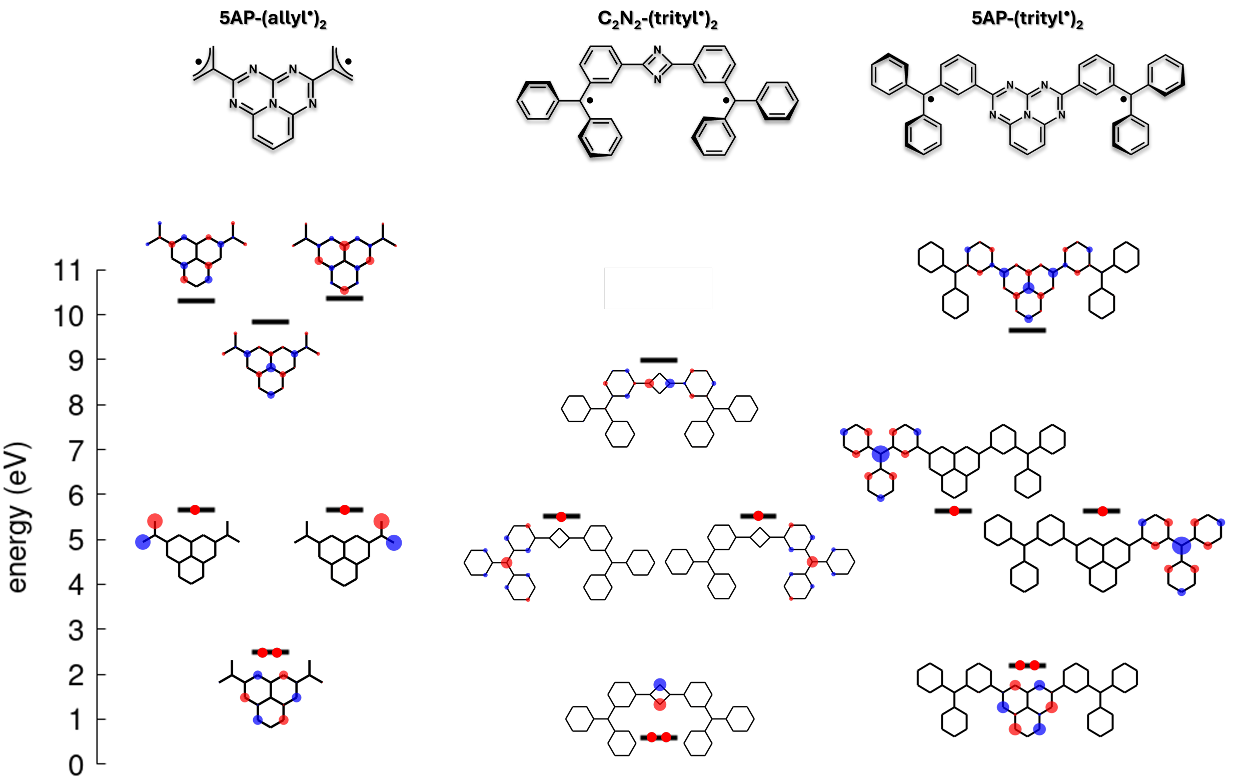

Figure S1: Frontier MOs calculated at the PPP-HF level and included in the RAS2 subsapce for 5AP-(allyl$^\bullet$)$_2$, C_2N_2-(trityl$^\bullet$)$_2$, and 5AP-(trityl$^\bullet$)$_2$, shown alongside their corresponding energy levels. Orbitals are marked with one or two red dots to indicate single or double occupancy, respectively. The PPP model parameters correspond to those reported in the captions of Figs. 2, 3, and 4 in the main text.

this method depends strongly on the choice of orbitals and the allocation of electrons across the RAS subspaces. In our case, we use a RAS2 space containing 4 electrons in 6 MOs for 5AP-(allyl$^\bullet$)$_2$, and 4 MOs for both C_2N_2-(trityl$^\bullet$)$_2$ and 5AP-(trityl$^\bullet$)$_2$. With these active spaces, the PPP-RASCI(h,p,hp) results are in close correspondence with high-level multireference *ab initio* benchmarks. The specific PPP-HF MOs included in RAS2 are shown in Fig. S1. For completeness, the full sets of PPP-HF molecular orbitals for the three systems are shown in Figs. S2, S3, S4, from which the orbitals assigned to the RAS1 and RAS3 subspaces can be identified.

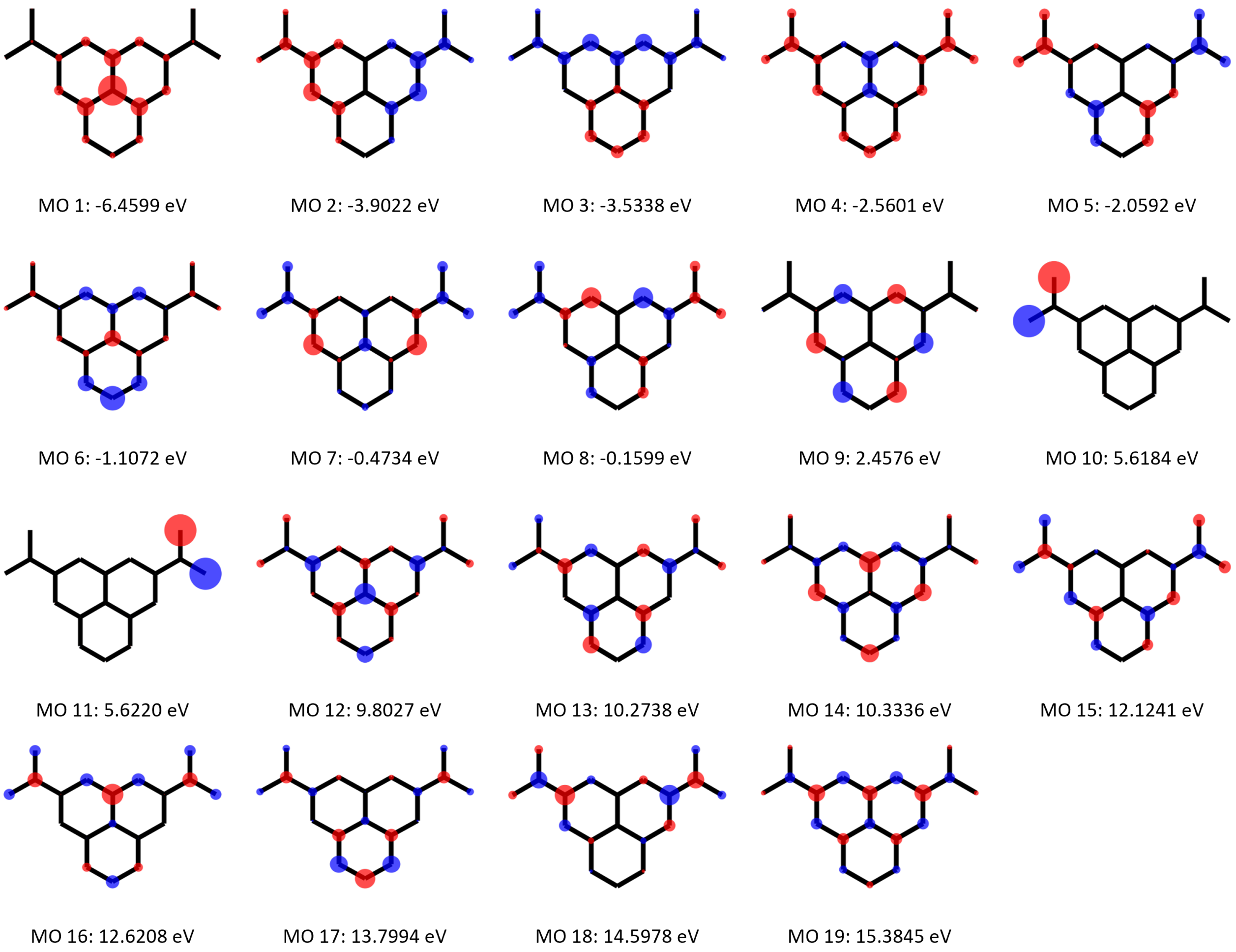

Figure S2: Energy levels calculated at the PPP-HF theory level for 5AP-(allyl$^\bullet$)$_2$ together with the relevant HF-MOs. MO 9 is the HOMO, MO 10 is SOMO$_1$, MO 11 is SOMO$_2$, and MO 12 is the LUMO. The PPP model parameters correspond to those reported in the caption of Fig. 2 in the main text.

S1.4 The RAS2 Space Dimension Dependence

To check the sensitivity of the PPP-RASCI results to the size of the RAS2 active space, we compared the results obtained using the RAS2 spaces adopted in the main text with those from smaller or larger RAS2 spaces. For 5AP-(allyl$^\bullet$)$_2$, in Fig. S5, we report results obtained with a reduced RAS2 space containing 4 electrons in 4 MOs. When using this smaller RAS2 space, the spin–spin exchange interaction triggered after photoexcitation is still present. However, both the S$_1$ and T$_1$ transition energies are slightly smaller than those obtained with the larger (4,6) RAS2 space. The ST energy gap increases by approximately

25 meV relative to the (4,6) case, while the spin–orbit coupling magnitude is roughly halved. Moreover, the characteristic SOC maximum observed at $\theta = \pm 30°$ with the (4,6) RAS2 space is no longer reproduced. These differences can be rationalized by considering that the two additional virtual orbitals included in the (4,6) RAS2 space lie only ∼0.5 eV above the LUMO and contribute to a more accurate description of the S_1 and T_1 states, resulting in a closer correspondence with the *ab initio* multireference benchmarks. In contrast, the deeper filled MOs, located at least 2.6 eV below the HOMO (see Fig. S2), play no significant role in the low-lying excitations and are therefore excluded from the RAS2 active space.

For the C_2N_2-bridged diradical, we also examined the effect of extending the RAS2 space by including one additional virtual orbital, corresponding to a (4,5) configuration (see Fig. S6). The results obtained for the (4,4) and (4,5) RAS2 spaces are closely matching, with negligible differences in both the excitation energies and the ST energy gap. The added virtual orbital, lying approximately 1.6 eV above the LUMO, does not play a major role in the description of the lowest singlet and triplet states. Similarly, the filled MOs lying below the HOMO are mainly localized on the trityl phenyl rings and do not contribute to the S_1 and T_1 states.

S1.5 The PPP SOMO-LUMO exchange integral

The $SOMO_1$-LUMO (equivalently $SOMO_2$-LUMO) exchange integral reads:

$$K_{SOMO_1-LUMO} = \frac{e^2}{4\pi\varepsilon_0}\left\langle \psi_{SOMO_1}(1)\psi_{LUMO}(2)\left|\frac{1}{r_{12}}\right|\psi_{LUMO}(1)\psi_{SOMO_1}(2)\right\rangle \quad \text{(S8)}$$

By expanding the MOs on the $2p_z$ basis and adopting the zero differential overlap approximation, Eq. S8 becomes:

$$K_{SOMO_1-LUMO} = \sum_{\mu\nu} c_{SOMO_1,\nu}c_{LUMO,\nu}c_{LUMO,\mu}c_{SOMO_1,\mu}V_{\nu\mu} \quad \text{(S9)}$$

where the double sum runs over the atomic orbitals, $c_{SOMO_1,\nu}$ is the coefficient of ν atomic orbital on the SOMO$_1$ ($c_{LUMO,\nu}$ is the same for LUMO) and $V_{\nu\mu}$ is the electrostatic repulsion between electrons on sites μ and ν, as introduced in Eq. S1. Looking at Eq. S8, the exchange energy becomes zero when SOMO and LUMO orbitals are disjoint, though it can remain small even when some overlap is present.

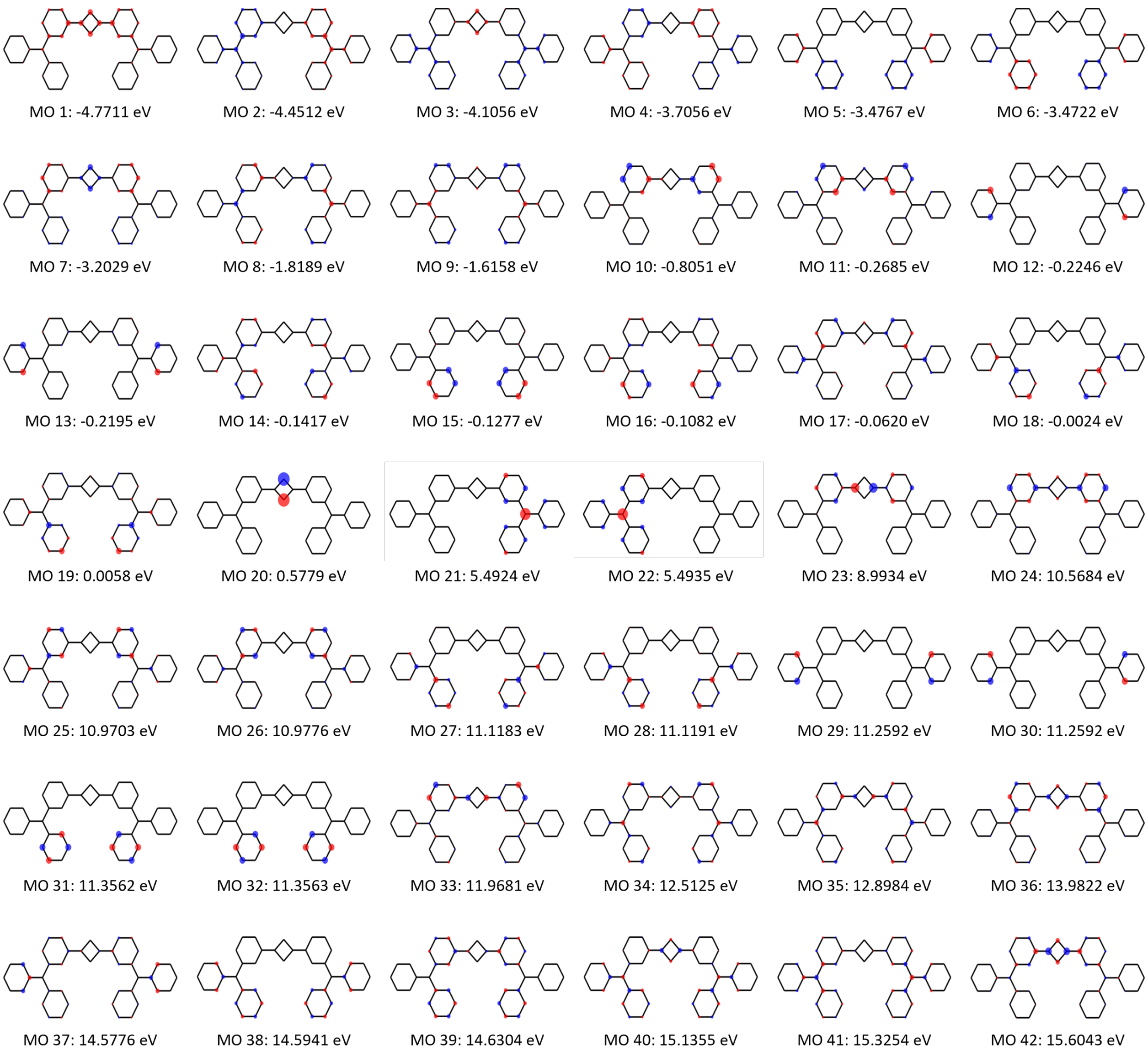

Figure S3: Energy levels calculated at the PPP-HF theory level for C_2N_2-(trityl$^\bullet$)$_2$ together with the relevant HF-MOs. MO 20 is the HOMO, MO 21 is $SOMO_1$, MO 22 is $SOMO_2$, and MO 23 is the LUMO. The PPP model parameters correspond to those reported in the caption of Fig. 3 in the main text.

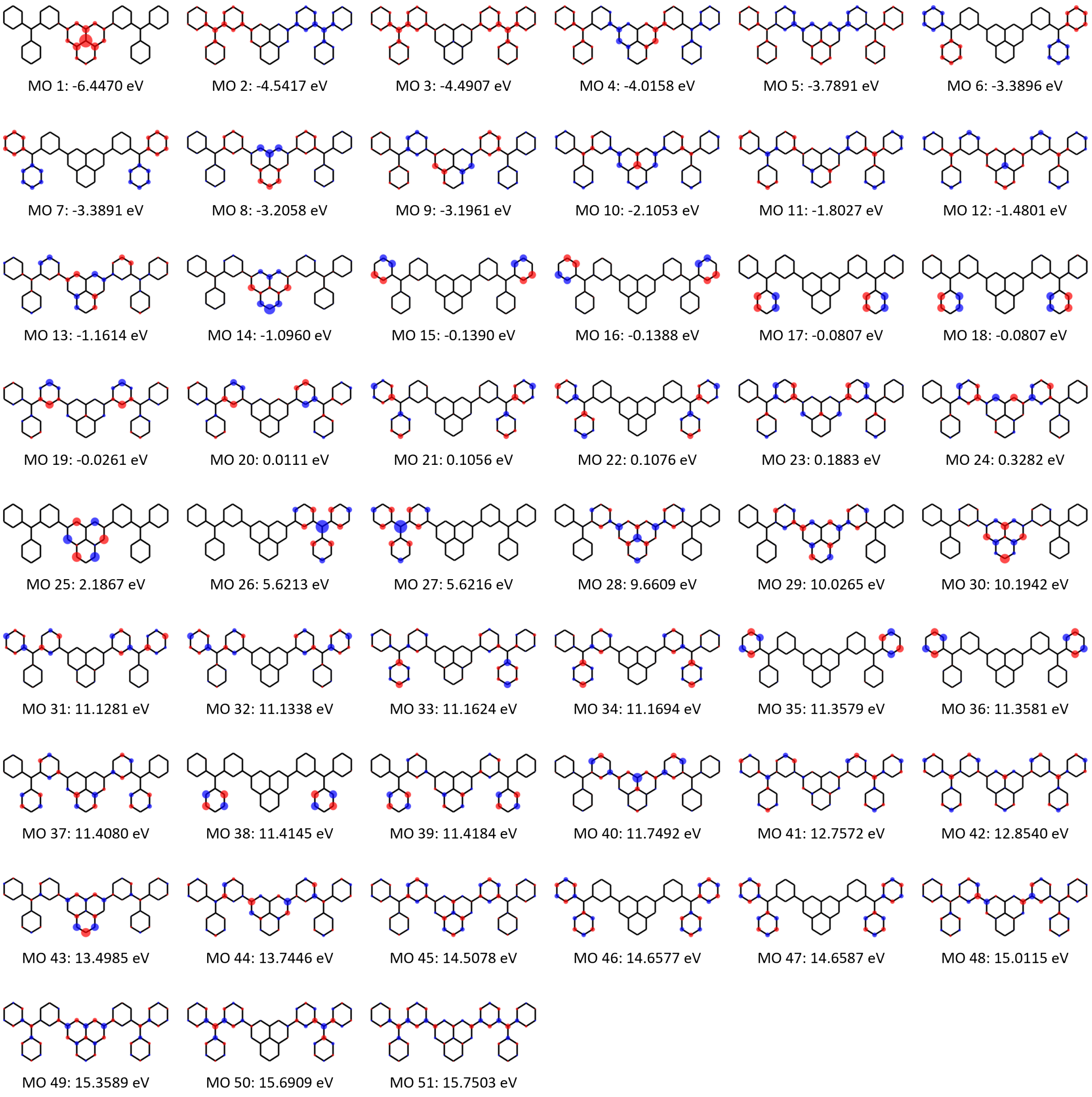

Figure S4: Energy levels calculated at the PPP-HF theory level for 5AP-(trityl$^\bullet$)$_2$ together with the relevant HF-MOs. MO 25 is the HOMO, MO 26 is SOMO$_1$, MO 27 is SOMO$_2$, and MO 28 is the LUMO. The PPP model parameters correspond to those reported in the caption of Fig. 4 in the main text.

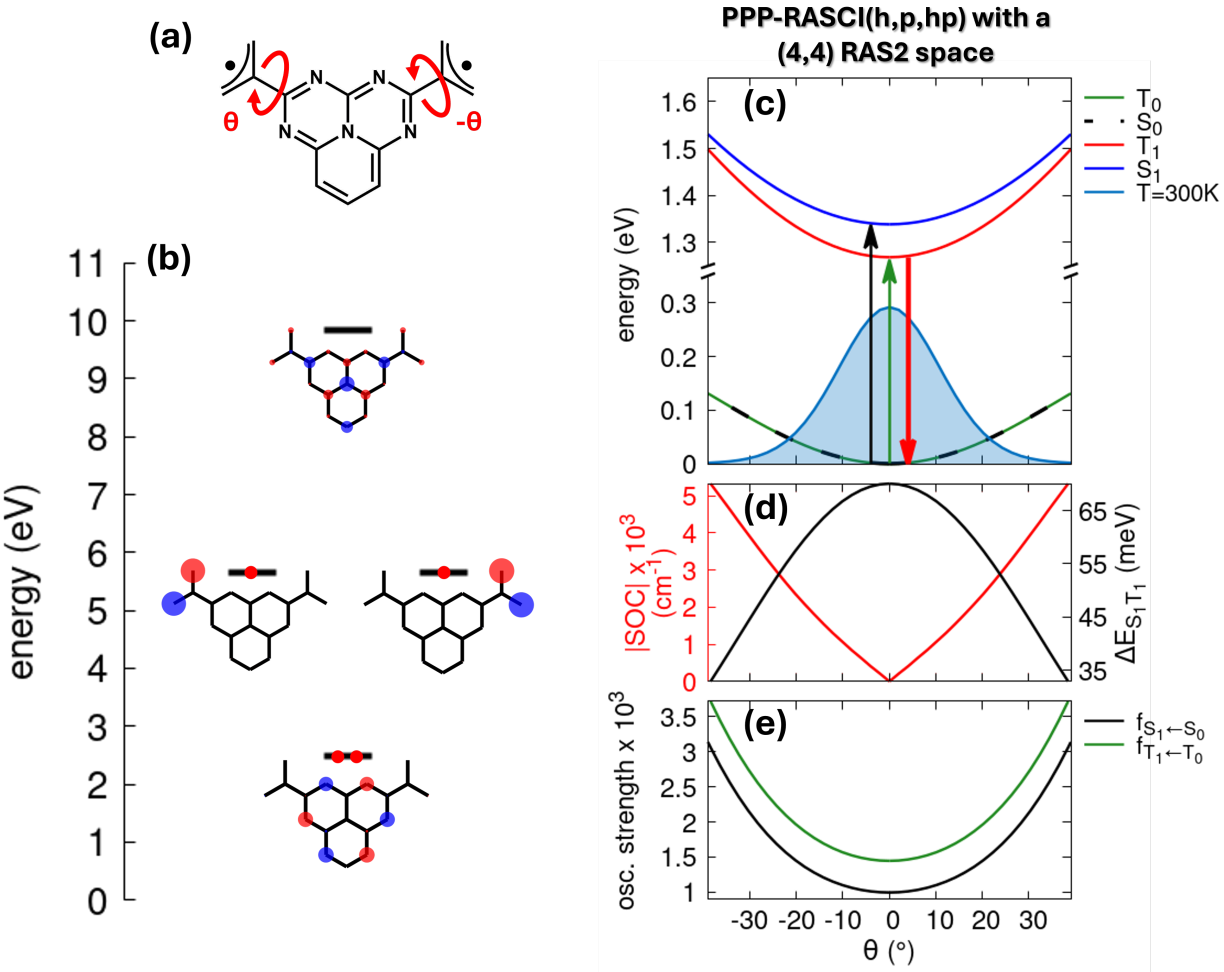

Figure S5: Electronic structure and photophysical properties of the 5AP-(allyl$^\bullet$)$_2$. (a) Molecular structure with torsional coordinate θ around the bridge-radical connecting bonds. (b) PPP-HF frontier MOs entering the RAS2 space at $\theta = 0°$. (c) PPP potential energy curves for S_0, T_0, S_1, T_1 states and ground state Boltzmann distribution at room temperature. (d) Singlet-triplet energy gap (black) and spin-orbit coupling magnitude (red) vs θ. (e) Oscillator strengths for $S_0 \rightarrow S_1$ (black) and $T_0 \rightarrow T_1$ (green) transitions. PPP calculations used the RASCI(h,p,hp) approach with a (4,4) RAS2 space. The PPP model parameters correspond to those reported in the caption of Fig. 2 in the main text.

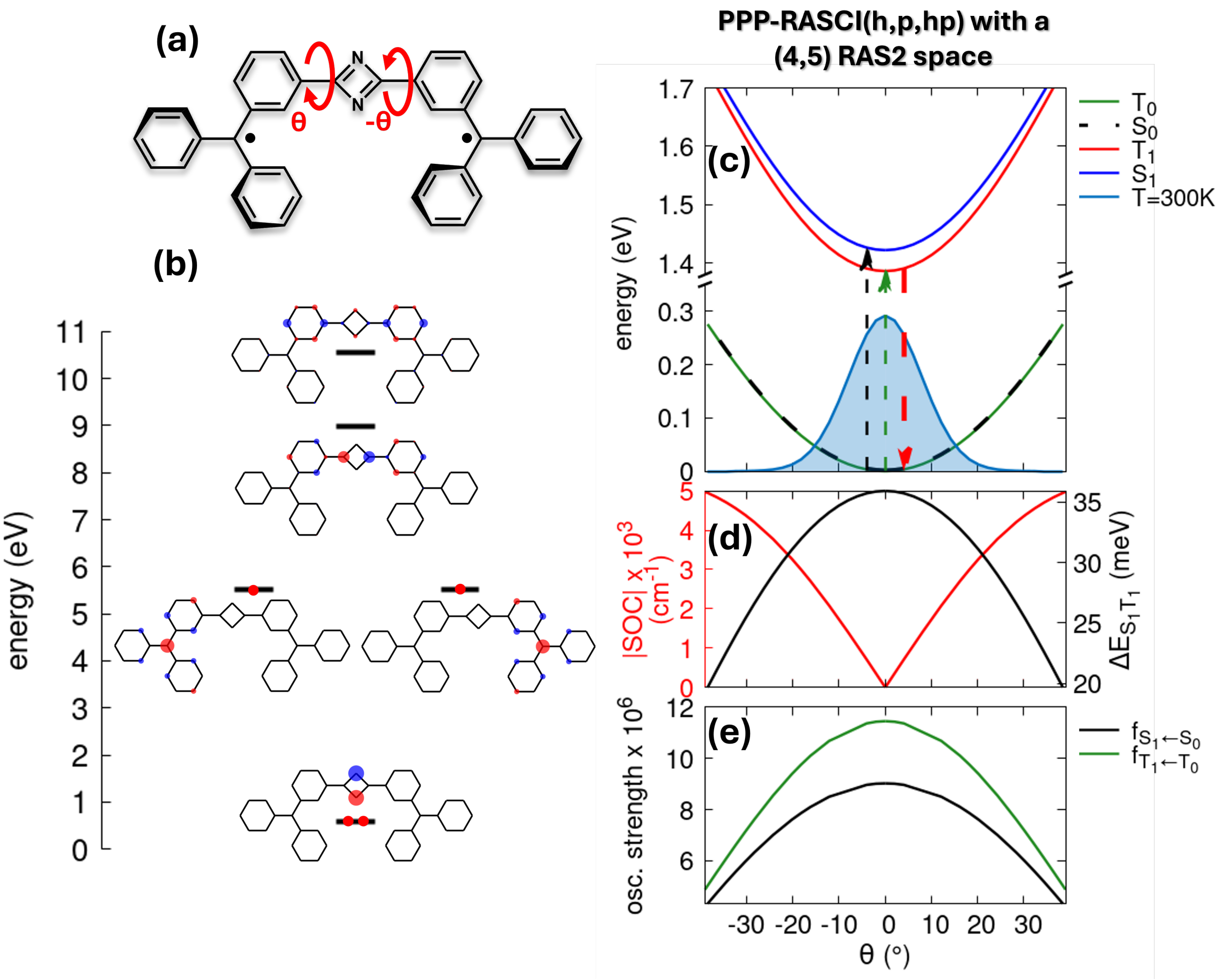

Figure S6: Electronic structure and photophysical properties of C_2N_2-(trityl$^{\bullet}$)$_2$. (a) Molecular structure showing the trityl-based C_2N_2-diradical system with torsional angle θ around the bridge-radical bonds. (b) PPP-HF frontier MOs entering the RAS2 space at $\theta = 0°$. (c) PPP potential energy surfaces and ground state thermal distribution at room temperature. (d) Torsional dependence of the S_1-T_1 energy gap (black) and spin-orbit coupling strength (red). (e) Oscillator strengths for $S_0 \rightarrow S_1$ (black) and $T_0 \rightarrow T_1$ (green). PPP calculations used the RASCI(h,p,hp) approach with a (4,5) RAS2 space. The PPP model parameters correspond to those reported in the caption of Fig. 3 in the main text.

S2 *Ab initio* Computational Details

S2.1 Computational Setup and Electronic Structure Methods

Geometry optimizations for the InveST-bridged diradicals depicted in Fig.1d in the main text were carried out at the DFT level using the UBHandHLYP functional[25,26] and the 6-31++G(d,p) basis set in the gas phase. All structures were optimized in their triplet ground states. To explore the conformational landscape, potential energy surface scans were performed by rigidly rotating the torsional angle θ around the two bonds linking the InveST core to the radical units, using 2° increments. The resulting energy profiles were then fitted to the steric potential function $V_{\text{steric}}(\theta)$. For all investigated systems, the equilibrium torsional angle was found to be $\theta_{\text{eq}} = 0°$ for both S_0/T_0 and S_1/T_1. This result was confirmed by excited-state geometry optimizations performed at the TD-DFT level (CAM-B3LYP/def2-SVP) for both S_1 and T_1 (see Section S7). For the excited-state geometry optimizations, we used CAM-B3LYP because its range-separated character provides a more reliable description of the multi-resonant charge-transfer S_1 and T_1 states of the InveST bridge. The ground-state geometry was optimized with 6-31++G(d,p) to better capture the diffuse spin density, while the excited states were optimized with the more compact def2-SVP basis for computational efficiency. This difference does not affect the structural conclusions: ground-state optimizations performed with def2-SVP differ from those with 6-31++G(d,p) by less than 0.002Å in bond lengths and 0.3° in bond angles, and both predict an equilibrium torsional angle of $\theta = 0°$. Ground state geometry optimizations were carried out using the Gaussian16 software package,[27] while excited state geometry optimizations were carried out using the Orca package (version 5.0.3).[28]

CASSCF calculations were carried out on the UBHandHLYP-optimized triplet ground-state geometries. To incorporate dynamic electron correlation, the CASSCF state energies were further refined using the van Vleck quasi-degenerate (QD) extension to strongly contracted second-order N-electron valence state perturbation theory (SC-NEVPT2).[29,30] All

the CASSCF/QD-NEVPT2 calculations were done with the Orca package (version 5.0.3).[28] CASSCF calculations were performed using two different active spaces: (4,4) and (6,6), with results for the latter reported in Section S2.2. All calculations employed the def2-SVP basis set, along with the Resolution of Identity (RI) approximation using the def2/JK auxiliary basis set. The first two triplet and the first two singlet roots were computed in each case. The (4,4) active space included four electrons in four frontier MOs, while the (6,6) space comprised six electrons in six MOs. The MOs obtained from CASSCF(4,4)/def2-SVP and CASSCF(6,6)/def2-SVP show minimal differences. Second-order perturbative corrections were generally modest across all systems for both (4,4) and (6,6) active spaces. For 5AP-$(\text{allyl}^{\bullet})_2$, corrections ranged from approximately 0.6 eV for T_1 to 0.8 eV for S_1. In contrast, significantly smaller corrections were observed for C_2N_2-$(\text{trityl}^{\bullet})_2$ (~ 0.1 eV for both S_1 and T_1) and 5AP-$(\text{trityl}^{\bullet})_2$ (~ 0.07 eV for both states).

S2.2 Active Space Dependence: QD-NEVPT2 Energies and CASSCF Molecular Orbitals

We present results obtained using a larger (6,6) active space, extending the (4,4) configuration used in the main text. Specifically, CASSCF molecular orbitals are shown for two representative θ values: 0° and 30°. Upon enlarging the active space, the key frontier orbitals – HOMO, SOMO, and LUMO – remain largely unchanged for both 5AP-$(\text{allyl}^{\bullet})_2$ and C_2N_2-$(\text{trityl}^{\bullet})_2$ systems, as shown in Figures S7 and S8. Total energies of the singlet and triplet states also follow similar trends, with S_0 and T_0 staying degenerate and T_1 consistently lying below S_1. For 5AP-$(\text{allyl}^{\bullet})_2$ system at $\theta = 0°$, the S_1-T_1 energy gap slightly increases from 25 meV with (4,4) to 42 meV with (6,6). At $\theta = 30°$, however, both active spaces yield nearly identical results. For the C_2N_2-$(\text{trityl}^{\bullet})_2$ system, the S_1–T_1 energy gap obtained with (4,4) and (6,6) active spaces perfectly match at both torsional angles.

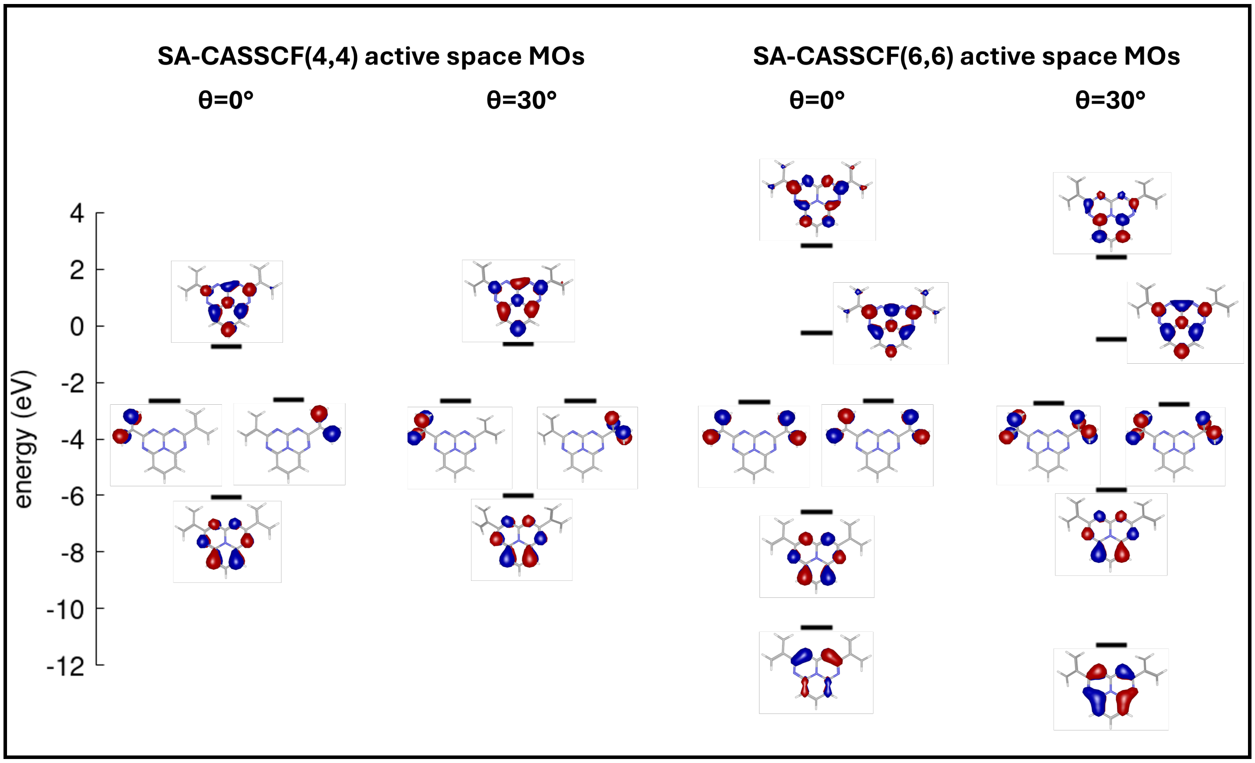

Figure S7: CASSCF frontier molecular orbitals of 5AP-(allyl$^\bullet$)$_2$ calculated with (4,4) and (6,6) active spaces using the def2-SVP basis set at $\theta = 0°$ and $\theta = 30°$ torsional angles.

Table S2: Total energies in atomic units of the S_0, T_0, T_1, and S_1 states of 5AP-(allyl$^\bullet$)$_2$ for planar ($\theta = 0°$) and twisted ($\theta = 30°$) geometries at the QD-NEVPT2 theory level.

	QD-NEVPT2(4,4)	QD-NEVPT2(6,6)	QD-NEVPT2(4,4)	QD-NEVPT2(6,6)
	$\boldsymbol{\theta = 0°}$		$\boldsymbol{\theta = 30°}$	
$E(S_0)$	-810.675922	-810.678782	-810.673568	-810.677085
$E(T_0)$	-810.675935	-810.678649	-810.673605	-810.676885
$E(T_1)$	-810.612415	-810.613130	-810.608965	-810.605813
$E(S_1)$	-810.611506	-810.611599	-810.608621	-810.605831

Table S3: Total energies in atomic units of the S_0, T_0, T_1, and S_1 states of C_2N_2-(trityl$^\bullet$)$_2$ for planar ($\theta = 0°$) and twisted ($\theta = 30°$) geometries at the QD-NEVPT2 theory level. Results are shown for two different active spaces.

	QD-NEVPT2(4,4)	QD-NEVPT2(6,6)	QD-NEVPT2(4,4)	QD-NEVPT2(6,6)
	$\boldsymbol{\theta = 0°}$		$\boldsymbol{\theta = 30°}$	
$E(S_0)$	-1644.065430	-1644.045811	-1644.059929	-1644.040222
$E(T_0)$	-1644.065446	-1644.045783	-1644.059936	-1644.040206
$E(T_1)$	-1643.995663	-1643.978743	-1643.988494	-1643.972841
$E(S_1)$	-1643.993669	-1643.976775	-1643.986834	-1643.971281

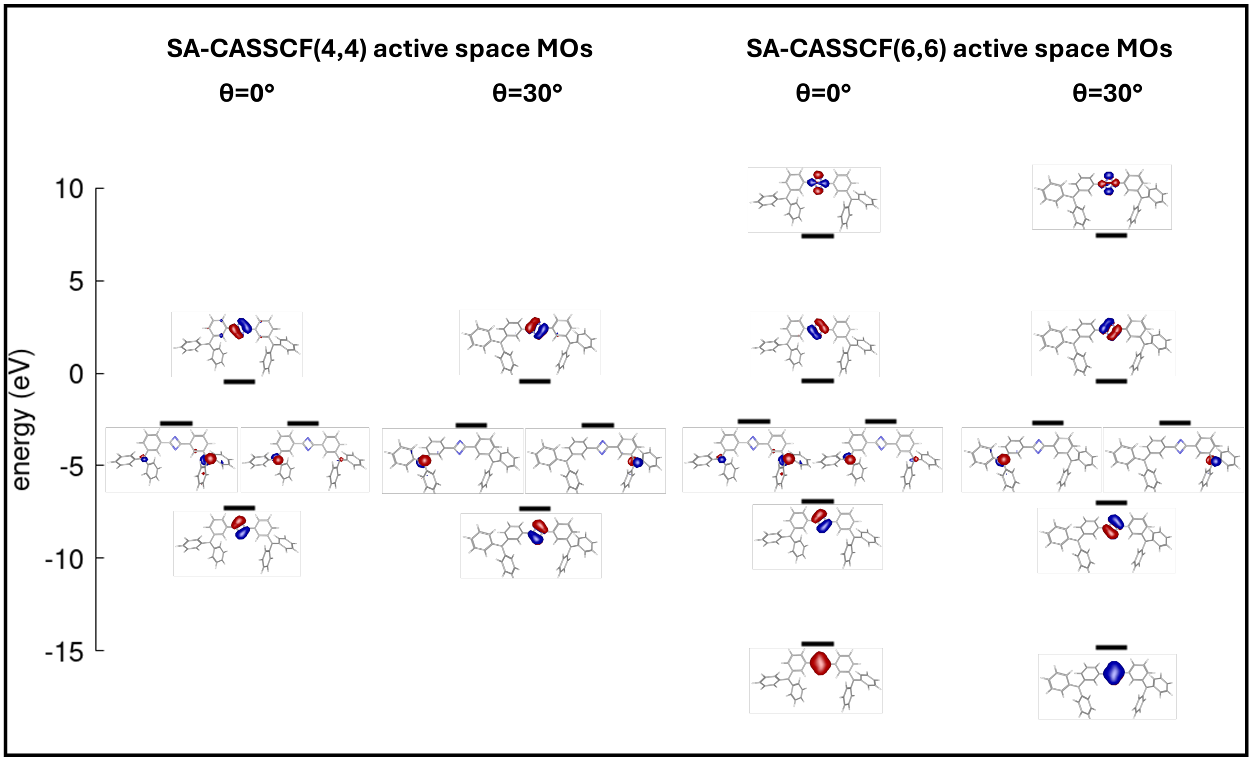

Figure S8: CASSCF frontier molecular orbitals of C_2N_2-(trityl$^{\bullet}$)$_2$ calculated with (4,4) and (6,6) active spaces using the def2-SVP basis set at $\theta = 0°$ and $\theta = 30°$ torsional angles.

S3 Electronic Character of S_1 and T_1 Excited States

The electronic character of the S_1 and T_1 states of the three InveST-bridged diradicals was examined by analyzing the weight of the most relevant determinants in the PPP and QD-NEVPT2 wavefunctions. In Fig.S9, we followed the relative weight of the leading open-shell diradical configuration and of the SOMO–SOMO charge-transfer configuration along the torsional coordinate. Across the entire torsional range, both S_1 and T_1 are consistently dominated by the open-shell diradical configuration, while the SOMO–SOMO charge-transfer contribution remains negligible. To further quantify this observation, we also analyzed the CI coefficients of the leading configurations in the CASSCF(6,6)/QD-NEVPT2 wavefunctions for the three systems at representative torsional angles ($\theta = 0°$ and $30°$). In all cases, both S_1 and T_1 are largely dominated by the open-shell HOMO–LUMO diradical configuration. For 5AP-(allyl$^\bullet$)$_2$, the weight of the HOMO–LUMO diradical configuration is 0.82 in T_1 and 0.85 in S_1 at $\theta = 0°$, increasing to 0.90 for both states at $\theta = 30°$. In the C_2N_2-(trityl$^\bullet$)$_2$ system, the corresponding weights are 0.91 (T_1) and 0.90 (S_1) at $\theta = 0°$, and 0.88 (T_1) and 0.90 (S_1) at $\theta = 30°$. For the 5AP-(trityl$^\bullet$)$_2$ diradical, the HOMO–LUMO diradical configuration is nearly pure, with a coefficient of 0.99 for both S_1 and T_1 at both $\theta = 0°$ and $30°$. Also here, for all systems, the contribution from the SOMO–SOMO charge-transfer configuration remains negligible throughout the torsional coordinate.

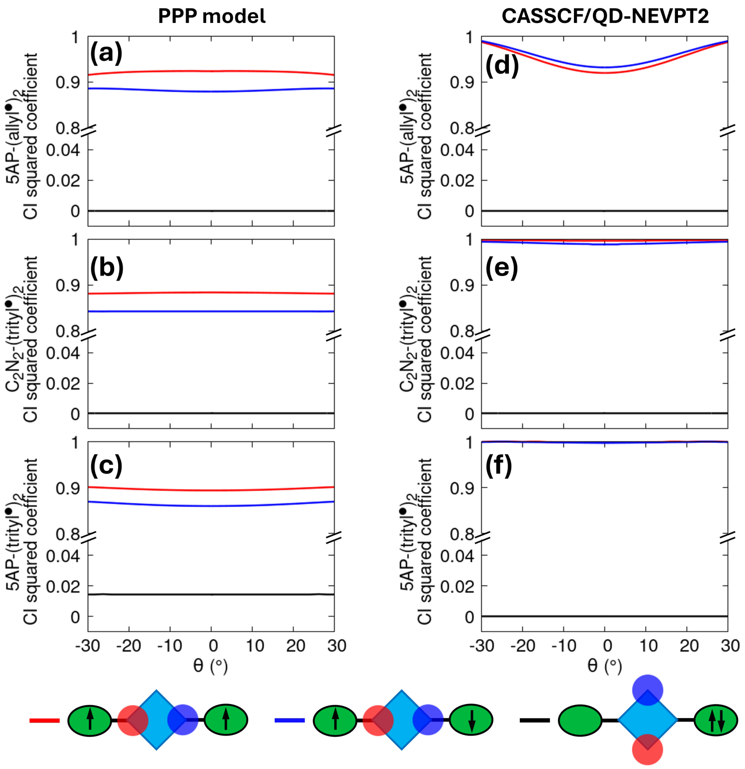

Figure S9: Relative contributions (squared CI coefficients) of selected excited-state configurations as a function of the torsional angle θ. Panels (a–c): PPP results for (a) 5AP-(allyl$^\bullet$)$_2$, (b) C_2N_2-(trityl$^\bullet$)$_2$, and (c) 5AP-(trityl$^\bullet$)$_2$. Panels (d–f): corresponding CASSCF(4,4)/QD-NEVPT2 results. The contributions of the HOMO–LUMO diradical configuration in T_1 (red) and S_1 (blue), as well as the SOMO–SOMO charge-transfer configuration in S_1 (black) are shown. PPP model parameters are the same as in the main text.

S4 Higher-lying excited states in C_2N_2-(trityl$^\bullet$)$_2$

We present a detailed analysis of the vertical excitation energies of C_2N_2-(trityl$^\bullet$)$_2$ at $\theta = 0°$. This system was chosen among the studied diradicals because the symmetric C_2N_2 bridge leads to dark S_1 and T_1 states, so excitation occurs through higher-lying states. Calculations were performed at both the PPP and CASSCF/QD-NEVPT2 levels of theory. For the PPP calculations, we used the RASCI(h,p,hp) approach with the same RAS2 space employed in Figure 3 of the main text. For the CASSCF calculations, we employed an active space of eight electrons distributed in eight molecular orbitals, (8,8), computing the first five singlet and five triplet roots. The active space was constructed to properly describe the high-lying excited states involving the radical units and includes two bonding and two antibonding molecular orbitals belonging to the trityl radicals, together with the two SOMOs and the HOMO and LUMO of the InveST bridge. The CASSCF(8,8) molecular orbitals are shown in Figure S10. Second-order perturbative corrections were generally modest, ranging from 0.7 to 0.8 eV.

The computed vertical excitation energies are reported in Table S4. At the PPP level, the S_2 state lies at 1.84 eV, only 0.36 eV above S_1. This state is a doubly excited state described by the HOMO/SOMO→SOMO/LUMO double excitation and is consequently optically dark. The first bright singlet state is S_3 at 2.47 eV (0.99 eV above S_1), which corresponds to a SOMO→LUMO transition. In the triplet manifold, T_2 at 1.69 eV is an optically dark state corresponding to an excitation localized on the InveST bridge (HOMO→LUMO). The T_3 state at 1.80 eV is also dark, arising from the HOMO/SOMO→SOMO/LUMO double excitation. The first bright triplet state is T_4 at 2.34 eV, corresponding to a SOMO→LUMO transition analogous to that found for S_3. The CASSCF/QD-NEVPT2 calculations provide qualitative confirmation of this electronic structure picture, with the same ordering and character of the excited states.

These results demonstrate that photoexcitation can occur through the bright S_0→S_3 and T_0→T_4 transitions, both involving the radical SOMOs. The presence of intermediate dark

states (S_2 in the singlet manifold and T_2, T_3 in the triplet manifold) at lower energies provides a cascade of states through which the system can relax via internal conversion to S_1 and T_1 states.

Table S4: Transition energies (in eV) for the first three excited singlet states and four excited triplet states of C_2N_2-(trityl$^\bullet$)$_2$ in the planar geometry ($\theta = 0°$), computed at the PPP-RASCI(h,p,hp) and CASSCF(8,8)/QD-NEVPT2 levels of theory. Corresponding oscillator strengths are given in parentheses.

	PPP-RASCI(h,p,hp)	CASSCF(8,8)/QD-NEVPT2
	**$\theta = 0°$**	
$E(S_1)$	1.48 (0.0000)	1.98 (0.0000)
$E(S_2)$	1.84 (0.0001)	2.22 (0.0002)
$E(S_3)$	2.47 (0.0224)	2.75 (0.0038)
$E(T_1)$	1.44 (0.0000)	1.89 (0.0000)
$E(T_2)$	1.69 (0.0000)	1.95 (0.0000)
$E(T_3)$	1.81 (0.0000)	2.22 (0.0002)
$E(T_4)$	2.39 (0.0209)	2.79 (0.0043)

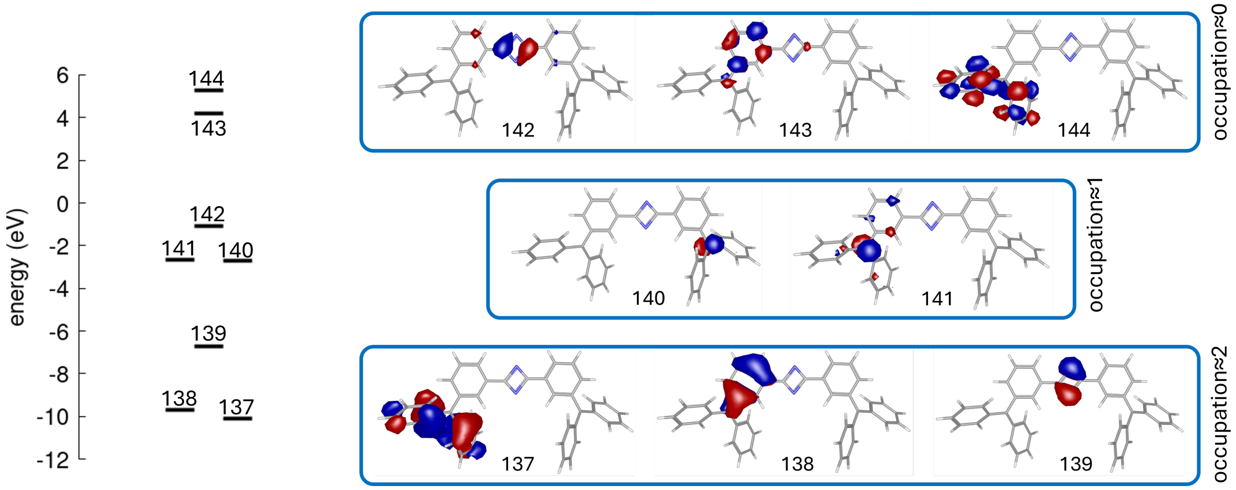

Figure S10: CASSCF frontier molecular orbitals of C_2N_2-(trityl$^\bullet$)$_2$ calculated with an (8,8) active space and the def2-SVP basis set at the planar geometry ($\theta = 0°$). These orbitals constitute the active space used to describe the higher-lying excited states in C_2N_2-(trityl$^\bullet$)$_2$.

S5 Torsional Angle Dependence in 5AP-(trityl$^\bullet$)$_2$

The torsional potential energy curves of 5AP-(trityl$^\bullet$)$_2$ were studied at the PPP-RASCI(h,p,hp) level using a (4,4) active space (4 electrons in 4 MOs, shown in Fig. S11a). Due to the high computational cost associated with QD-NEVPT2 calculations for this large system, they were performed only at selected torsional angles ($\theta = 0°$ and 30°), rather than across the full angular range sampled with the PPP model. To evaluate the influence of active space size, comparisons between the (4,4) and (6,6) active spaces were performed at these selected geometries (see Table S5), with the corresponding CASSCF MOs shown in Fig. S12.

As in the smaller diradical systems, the potential energy curves (panel c) show that the ground state manifold adopts a planar equilibrium geometry at $\theta = 0°$. Moreover, the singlet (S_0) and triplet (T_0) states remain degenerate throughout the entire torsional range, confirming the expected lack of interaction between the radical centers in the ground state. In the excited-state manifold, an energy gap still opens between the first excited triplet (T_1) and singlet (S_1) states, with T_1 lying below S_1. However, the magnitude of this gap is strongly reduced compared to the smaller diradical systems. At $\theta = 0°$, the S_1–T_1 gap reaches just 17 meV at the PPP-RASCI(h,p,hp) level (black curve in panel d) and only 5 meV at the QD-NEVPT2 level (see Table S5), highlighting the weakened spin–spin coupling resulting from the extended through-bond separation between the radical centers. This already small energy gap diminishes further as the torsional angle increases.

The SOC between S_1 and T_1 is similarly reduced. The maximum absolute value of the SOC matrix element is 4.5×10^{-4} cm^{-1} at the PPP-RASCI(h,p,hp) level (red curve in panel d) and 10^{-3} cm^{-1} at the QD-NEVPT2 level, nearly two orders of magnitude smaller than the values found for the 5AP-(allyl$^\bullet$)$_2$ system (cf. Fig.2d, g in the main text).

To further assess the sensitivity of the results to the active-space size, additional PPP-RASCI(h,p,hp) calculations were performed using an enlarged RAS2 space containing 4 electrons in 6 orbitals, obtained by adding two virtual orbitals (MOs 29 and 30 in Fig. S4) lying approximately 0.37 eV above the LUMO to the (4,4) RAS2 space. The S_1 and T_1

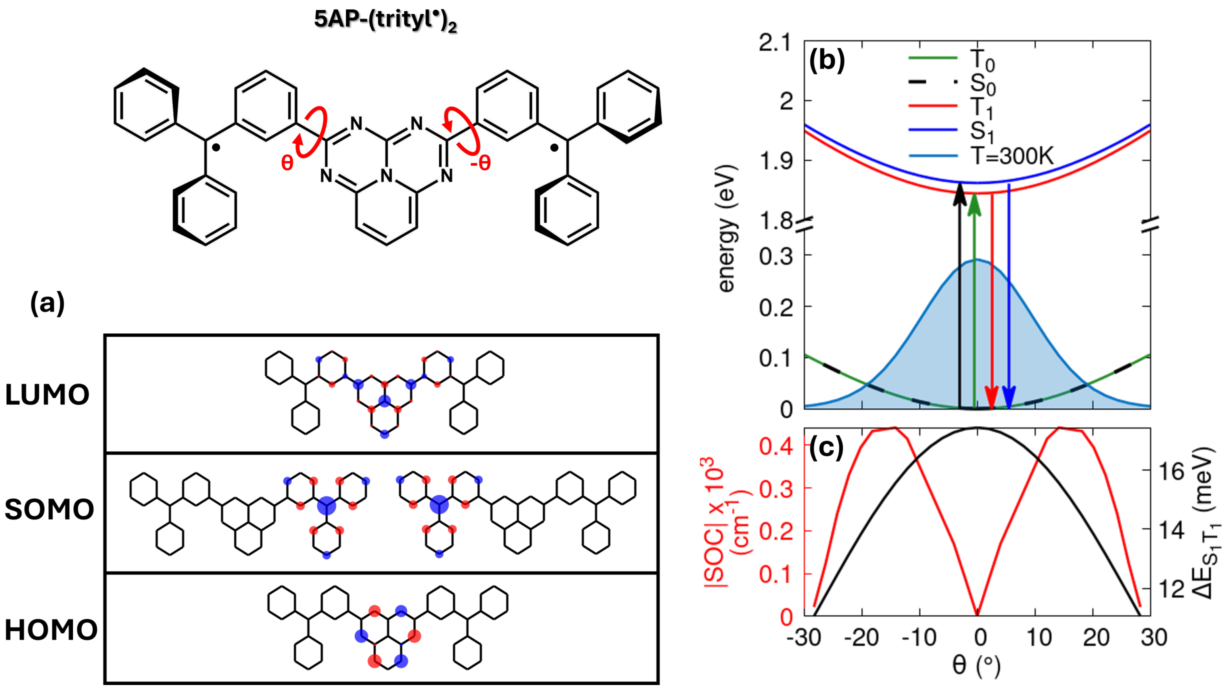

Figure S11: Electronic structure of the trityl-based 5AP-diradical system at the PPP-RASCI(h,p,hp) theory level. (a) PPP frontier molecular orbitals entering RAS2 subspace at planar ($\theta = 0°$) geometry. (b) PPP potential energy curves and ground state thermal distribution. (c) Torsional dependence of the S_1-T_1 energy gap (black) and spin-orbit coupling strength (red). The PPP model parameters correspond to those reported in the captions of Fig. 4 in the main text.

transition energies computed with the enlarged (4,6) RAS2 space are approximately 0.15 eV higher than those from the (4,4) case, while the ST gap is 14 meV smaller (see Table S6). The SOC magnitude obtained with the (4,6) RAS2 space is 0.007 cm^{-1} at $\theta = 30°$. Because of the high computational cost associated with the enlarged RAS2, these calculations were carried out only for $\theta = 0°$ and $\theta = 30°$, instead of over the full torsional range considered for the (4,4) case. The problem dimension for the largest spin manifold ($S_z = 0$) increases from 59,700 basis determinants for the (4,4) RAS2 to 366,165 for the (4,6) RAS2 space.

Table S5: Total energies in atomic units of the S_0, T_0, T_1, and S_1 states of 5AP-(trityl$^\bullet$)$_2$ for planar ($\theta = 0°$) and twisted ($\theta = 30°$) geometries at the QD-NEVPT2 theory level. Results are shown for two different active spaces.

	QD-NEVPT2(4,4)	QD-NEVPT2(6,6)	QD-NEVPT2(4,4)	QD-NEVPT2(6,6)
	**$\theta = 0°$**		**$\theta = 30°$**	
$E(S_0)$	-2037.414432	-2037.412602	-2037.411533	-2037.417212
$E(T_0)$	-2037.414443	-2037.412421	-2037.411546	-2037.417112
$E(T_1)$	-2037.308775	-2037.312054	-2037.306158	-2037.321103
$E(S_1)$	-2037.308582	-2037.311809	-2037.305967	-2037.320838

Table S6: Total energies (in eV) of the S_0, T_0, T_1, and S_1 states, as well as the ST gap, of 5AP-(trityl$^\bullet$)$_2$ for planar ($\theta = 0°$) and twisted ($\theta = 30°$) geometries at the PPP-RASCI(h,p,hp) level of theory with a (4,6) RAS2 space. The PPP model parameters correspond to those reported in the captions of Fig. 4 in the main text.

	PPP-RASCI(h,p,hp) with (4,6) RAS2	
	**$\theta = 0°$**	**$\theta = 30°$**
$E(S_0)$	-142.060849	-141.686256
$E(T_0)$	-142.060773	-141.686207
$E(T_1)$	-140.053143	-139.678088
$E(S_1)$	-140.050502	-139.674854
$\Delta E_{S_1T_1}$	0.003	0.003

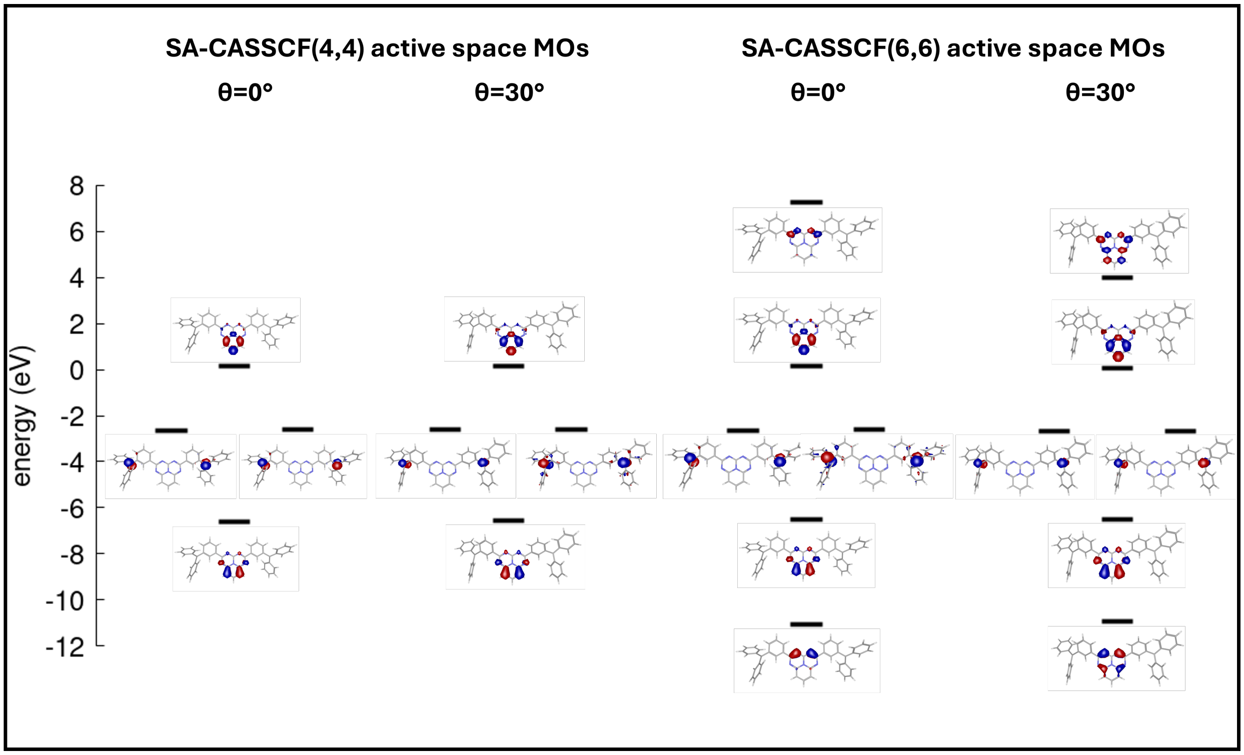

Figure S12: CASSCF frontier molecular orbitals of 5AP-(trityl$^{\bullet}$)$_2$ calculated with (4,4) and (6,6) active spaces using the def2-SVP basis set at $\theta = 0°$ and $\theta = 30°$ torsional angles.

S6 ISC and RISC Rate Calculation Details

S6.1 The Diabatization Procedure

To build the diabatic Hamiltonian used in the rate calculations (see Eq. 3 in the main text), a fitting procedure was carried out. The model parameters – τ_0, β_0, ω_t, a, $2z$, and $2s$ – were optimized to reproduce the adiabatic potential energy curves as a function of θ obtained from either PPP-RASCI or QD-NEVPT2 calculations. Since these two methods yield slightly different potential energy curves, independent diabatization procedures were performed for each dataset. During this fitting process, the SOC term V_{SOC} was set to zero. This approximation is justified by the small magnitude of SOC in these systems, which has a negligible effect on the shape of the adiabatic curves. Consequently, the diabatization was carried out independently for the singlet (S_0/S_1) and triplet (T_0/T_1) manifolds. For each manifold, the diabatic parameters were tuned so that the eigenvalues of the model closely matched the corresponding adiabatic curves across the entire range of θ. After completing the parameter fitting, the spin–orbit coupling term V_{SOC} – initially omitted – was reintroduced into the model. In the diabatic picture, V_{SOC} is assumed to be independent of θ and is treated as a constant. Its value was chosen so that the matrix element $|\langle S_1|V_{SOC}|T_1\rangle|$, computed using the eigenstates of the diabatic Hamiltonian, matches the θ-dependent SOC profile obtained from either the PPP-RASCI or QD-NEVPT2 calculations, depending on which dataset was used in the original fit.

S6.2 Quantum Treatment of the Torsional Degree of Freedom θ

With the diabatic model fully parametrized, we proceeded to compute the ISC (and RISC) rates. Due to the extremely small S_1–T_1 energy gap typical of InveST-bridged diradicals, an adiabatic treatment of the torsional coordinate is inadequate for accurately describing ISC dynamics. Instead, a fully quantum mechanical treatment of θ was adopted. To this end, the diabatic Hamiltonian was written on the basis obtained as the direct product of

the four electronic diabatic states times the eigenstates of the harmonic oscillator associated with the θ-dependent torsional potential. The dimensionless conformational coordinate can be written as:

$$\theta = (a^\dagger + a)/\sqrt{2} \tag{S10}$$

where $a^\dagger(a)$ is the bosonic creation (annihilation) operator. The infinite harmonic oscillator basis must be truncated to a large enough number of states to ensure convergence of the calculated quantities. Due to the very low frequency associated with the conformational mode, a large basis is required. In our case, 280 vibrational states were retained, resulting in a total Hamiltonian dimension of 560. Moreover, we used a polynomial expansion of $\cos 2\theta \sin 2\theta$, and, consistently with the quartic expansion of the potential, we truncated it at the third order:

$$\cos 2\theta \sin 2\theta \simeq 2\theta - \frac{8}{3}\theta^3 \tag{S11}$$

Diagonalizing the resulting vibronic Hamiltonian with $V_{SOC} = 0$ provides the singlet and triplet vibronic eigenstates used for rate calculations. Once these eigenstates are obtained, ISC and RISC rates are evaluated using Fermi Golden Rule.

The vibronic energy levels within the T_1 and S_1 manifolds, obtained by diagonalizing the vibronic Hamiltonian based on parameters fitted to the PPP-derived adiabatic potential energy curves, are shown as gray and blue lines in Fig. S13, panels a and c, respectively. The corresponding ISC and RISC rates, computed for various relaxation times τ, are displayed in panels b and d. To complement the ISC and RISC analysis, we also evaluated the radiative decay rates from the S_1 and T_1 states using PPP-derived transition energies and transition dipole moments. As observed in the main text when discussing the oscillator strength of 5AP-(allyl$^\bullet$)$_2$ as a function of the torsional angle θ (cf. Fig. 2e, main text), the PPP model underestimates transition dipole moments compared to multireference *ab initio*

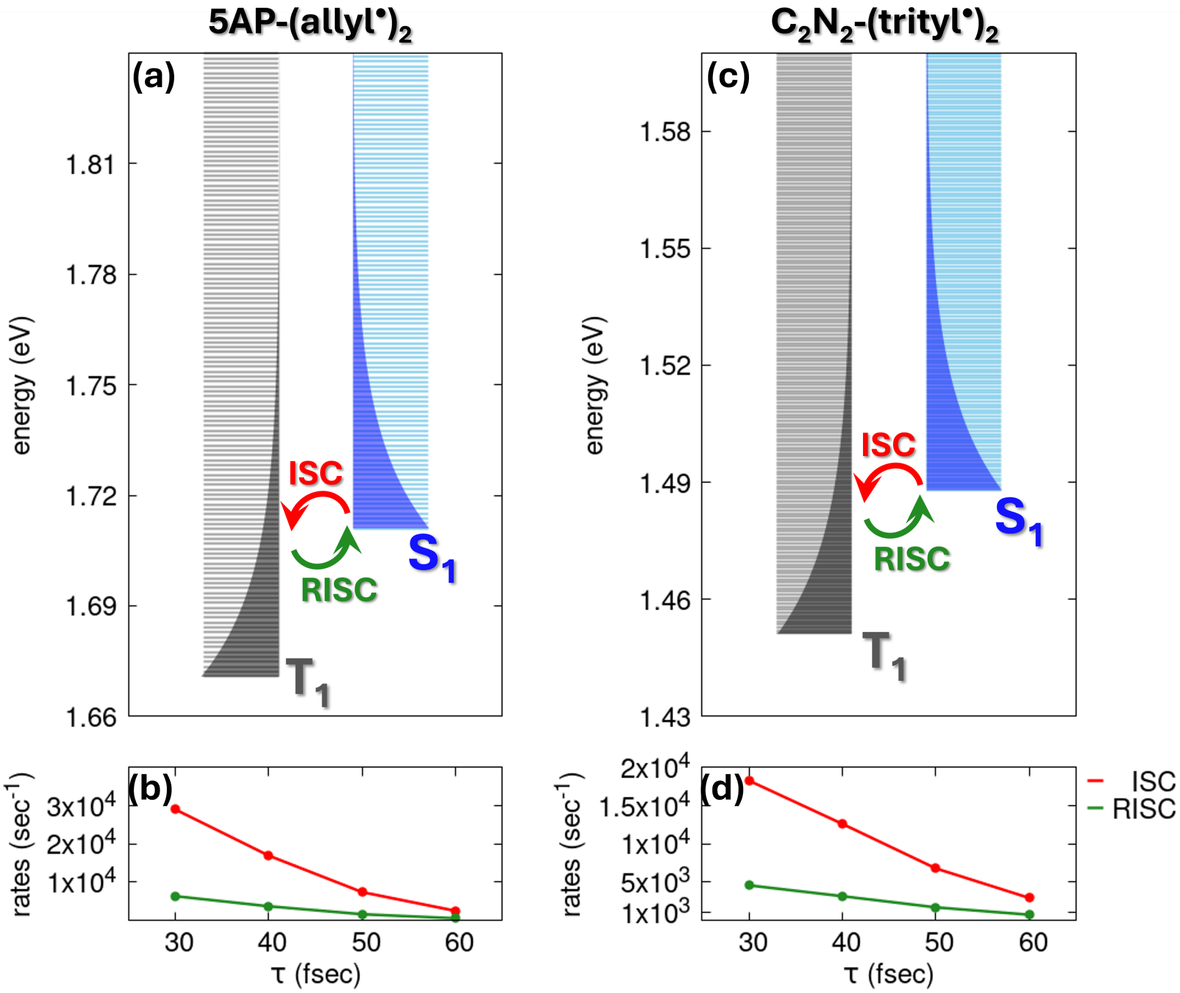

Figure S13: A schematic representation of the vibronic calculation of ISC rates and RISC rates for 5AP-(allyl$^\bullet$)$_2$ (panel a) and C_2N_2-(trityl$^\bullet$)$_2$ (panel c) starting from the PPP results shown in Figs. 2 and 3, main text. In both panels, gray and blue lines show the energy of the vibronic triplet and singlet eigenstates, respectively. The global ISC rate is calculated summing all the rates of the S_1 to T_1 processes, averaging on the thermal population of singlet states (graphically represented by the blue shaded area). RISC rates are evaluated from the ISC rate by imposing the microscopic reversibility condition. ISC and RISC rates calculated for different values of the relaxation time τ are reported in panels b and d. Parameters for 5AP-(allyl$^\bullet$)$_2$: $\tau_0 = 0.18$ eV, $\beta_0 = 0.21$ eV, $2z = 1.71$ eV, $2s = 1.67$ eV, $\hbar\omega_t = 1.3 \times 10^{-3}$ eV, $a = -0.15$ eV, $V_{SOC} = -0.09$ eV. Parameters for C_2N_2-(trityl$^\bullet$)$_2$: $\tau_0 = 0.20$ eV, $\beta_0 = 0.22$ eV, $2z = 1.49$ eV, $2s = 1.45$ eV, $\hbar\omega_t = 6.9 \times 10^{-4}$ eV, $a = -0.23$ eV, $V_{SOC} = -0.04$ eV.

methods (CASSCF/QD-NEVPT2). This discrepancy is reflected in the computed radiative rate, which amounts to 2.2×10^4 s^{-1} at the PPP level, in contrast to 3.5×10^7 s^{-1} from CASSCF/QD-NEVPT2 calculations. For the C_2N_2-(trityl$^\bullet$)$_2$ system, both S_1 and T_1 remain

optically dark due to the symmetric nature of the InveST core, leading to vanishing transition dipole moments and, consequently, negligible radiative rates even at the PPP level.

S7 Cartesian coordinates

Table S7: Cartesian coordinates for 5AP-(allyl$^\bullet$)$_2$ optimized geometry in the triplet ground state obtained at DFT level (UBHandHLYP/6-31++G(d,p)).

atom symbol	x (Å)	y (Å)	z (Å)
C	0.000000	-0.626272	-0.000005
C	-2.276848	-0.568055	-0.000002
C	-1.218291	1.441294	0.000036
C	0.000000	3.506509	0.000000
C	1.218291	1.441294	-0.000039
C	2.276849	-0.568055	0.000007
H	0.000000	4.584256	0.000001
C	-3.551681	-1.338940	-0.000009
C	3.551681	-1.338940	0.000011
N	0.000000	0.776006	-0.000002
C	3.486956	-2.721856	-0.000130
C	-3.486957	-2.721856	0.000130
H	-4.393984	-3.302043	0.000136
C	4.750607	-0.642788	0.000157
H	5.683927	-1.179355	0.000173
C	-4.750607	-0.642788	-0.000156
H	-5.683927	-1.179355	-0.000173
H	4.393984	-3.302043	-0.000137
H	2.542333	-3.232019	-0.000228
H	4.769666	0.430513	0.000257
H	-2.542333	-3.232019	0.000229
H	-4.769666	0.430513	-0.000255
N	1.147347	-1.274901	0.000048
N	2.359615	0.746180	-0.000069
N	-2.359615	0.746180	0.000071
N	-1.147347	-1.274901	-0.000049
C	-1.204545	2.830760	0.000038
H	-2.153629	3.332599	0.000065
C	1.204545	2.830760	-0.000038
H	2.153629	3.332599	-0.000063

Table S8: Cartesian coordinates for 5AP-(allyl$^\bullet$)$_2$ optimized geometry in the first excited triplet state T_1 obtained at TD-DFT level (CAM-B3LYP/def2-SVP).

atom symbol	x (Å)	y (Å)	z (Å)
C	-0.000000	-0.661603	-0.000005
C	2.303790	-0.604968	0.000018
C	1.241759	1.442800	0.000012
C	-0.000000	3.555063	-0.000002
C	-1.241759	1.442800	-0.000018
C	-2.303790	-0.604968	-0.000026
H	0.000000	4.648810	-0.000001
C	3.578735	-1.347305	0.000023
C	-3.578735	-1.347305	-0.000030
N	-0.000000	0.775699	-0.000004
C	-3.553153	-2.750490	-0.000025
C	3.553153	-2.750490	0.000023
H	4.490893	-3.315955	0.000024
C	-4.781363	-0.634111	-0.000032
H	-5.737489	-1.168264	-0.000031
C	4.781364	-0.634111	0.000025
H	5.737489	-1.168265	0.000026
H	-4.490892	-3.315955	-0.000024
H	-2.604693	-3.292051	-0.000022
H	-4.786438	0.457709	-0.000035
H	2.604693	-3.292051	0.000021
H	4.786438	0.457709	0.000025
N	-1.159966	-1.299914	-0.000016
N	-2.365383	0.767439	-0.000029
N	2.365383	0.767439	0.000022
N	1.159966	-1.299914	0.000007
C	1.212803	2.863932	0.000014
H	2.177933	3.372802	0.000026
C	-1.212803	2.863932	-0.000018
H	-2.177933	3.372802	-0.000028

Table S9: Cartesian coordinates for 5AP-(allyl$^{\bullet}$)$_2$ optimized geometry in the first excited singlet state S_1 obtained at TD-DFT level (CAM-B3LYP/def2-SVP.

atom symbol	x (Å)	y (Å)	z (Å)
C	-0.000000	-0.658252	-0.000004
C	2.288117	-0.562984	0.000012
C	1.245173	1.470580	0.000004
C	-0.000000	3.559811	-0.000001
C	-1.245173	1.470580	-0.000011
C	-2.288117	-0.562984	-0.000019
H	-0.000000	4.653391	0.000000
C	3.579346	-1.341603	0.000022
C	-3.579346	-1.341603	-0.000028
N	-0.000000	0.780103	-0.000003
C	-3.544056	-2.729376	-0.000027
C	3.544056	-2.729378	0.000025
H	4.471894	-3.312212	0.000032
C	-4.799189	-0.676340	-0.000036
H	-5.741022	-1.235696	-0.000042
C	4.799191	-0.676339	0.000026
H	5.741025	-1.235695	0.000034
H	-4.471893	-3.312209	-0.000032
H	-2.581578	-3.248640	-0.000020
H	-4.825113	0.416651	-0.000036
H	2.581575	-3.248642	0.000020
H	4.825114	0.416654	0.000023
N	-1.158358	-1.297119	-0.000012
N	-2.385086	0.758216	-0.000019
N	2.385086	0.758217	0.000013
N	1.158358	-1.297119	0.000003
C	1.217954	2.864455	0.000004
H	2.181289	3.377146	0.000010
C	-1.217955	2.864455	-0.000008
H	-2.181289	3.377146	-0.000013

Table S10: Cartesian coordinates for C_2N_2-(trityl$^{\bullet}$)$_2$ optimized geometry in the triplet ground state obtained at DFT level (UBHandHLYP/6-31++G(d,p)).

atom symbol	x (Å)	y (Å)	z (Å)
C	-0.829673	-2.192050	0.096349
N	0.028226	-1.224916	0.019404
N	0.089503	-3.339418	0.048163
C	0.947070	-2.371441	-0.029883
C	2.381071	-2.352227	-0.137771
C	-2.264343	-2.217317	0.200279
C	3.098837	-3.552877	-0.175202
C	3.034868	-1.127184	-0.196168
C	-2.982006	-1.022759	0.222603
C	-2.918465	-3.447365	0.271253
C	4.476625	-3.501806	-0.267656
C	4.434277	-1.060561	-0.285392
C	-4.302239	-3.465811	0.363642
C	-4.377143	-1.026506	0.311503
H	5.049415	-4.413614	-0.307933
C	5.133269	-2.279882	-0.318085
H	-4.826298	-4.404836	0.430374
C	-5.016475	-2.281453	0.379763
C	5.131797	0.217170	-0.337723
C	-5.143417	0.214448	0.332550
C	-4.595192	1.402753	0.976576
C	-3.783186	1.308085	2.119085
C	-4.860551	2.688322	0.475410
C	-3.267618	2.438243	2.727881
C	-4.336572	3.815862	1.081611
H	-2.654934	2.334319	3.608499
H	-4.544934	4.788246	0.665570
C	-3.538426	3.699466	2.212821
C	4.516478	1.362788	-0.999423
C	3.706870	1.204113	-2.136105
C	4.714078	2.669097	-0.521684
C	4.126870	3.755784	-1.144228
C	3.127634	2.293830	-2.761390
H	4.283795	4.745178	-0.746119
H	2.517774	2.141560	-3.636902
C	3.331327	3.576407	-2.269052
C	-6.461145	0.264030	-0.295448
C	-7.501372	1.030049	0.255135
C	-6.736284	-0.452251	-1.471674
C	-8.751494	1.071200	-0.336581
C	-7.984576	-0.401488	-2.066691

Table S10 – continued

atom symbol	x (Å)	y (Å)	z (Å)
H	-9.534778	1.657277	0.116223
H	-8.162309	-0.950690	-2.977093
C	-9.001575	0.357946	-1.502183
C	6.453003	0.350489	0.272131
C	6.778187	-0.317890	1.463443
C	7.444834	1.154906	-0.311338
C	8.028479	-0.185551	2.041422
C	8.697774	1.277361	0.263227
H	8.244814	-0.699355	2.964055
H	9.443940	1.891294	-0.214652
C	8.997883	0.610181	1.444056
H	-7.325732	1.579560	1.164372
H	-9.974403	0.394045	-1.964257
H	-5.954360	-1.033968	-1.930206
H	-5.465427	2.794370	-0.409008
H	-6.089318	-2.314913	0.464820
H	-2.340064	-4.355179	0.254740
H	-2.440383	-0.095087	0.150896
H	-3.572873	0.339388	2.539572
H	-3.131548	4.578620	2.684706
H	2.874996	4.423816	-2.753614
H	3.548886	0.218293	-2.539467
H	5.316729	2.822949	0.357250
H	7.229937	1.670375	-1.232020
H	2.446067	-0.227572	-0.149717
H	2.568029	-4.488844	-0.132546
H	6.206514	-2.264210	-0.402524
H	6.032975	-0.927054	1.946763
H	9.972504	0.709695	1.892871

Table S11: Cartesian coordinates for C_2N_2-(trityl$^\bullet$)$_2$ optimized geometry in the first excited triplet state T_1 obtained at TD-DFT level (CAM-B3LYP/def2-SVP).

atom symbol	x (Å)	y (Å)	z (Å)
C	-0.902157	-2.318858	0.084078
N	0.009677	-1.281190	0.008573
N	0.027852	-3.342714	0.044960
C	0.939708	-2.304744	-0.028697
C	2.364754	-2.295106	-0.112954
C	-2.326808	-2.332238	0.173109
C	3.081575	-3.509453	-0.124137
C	3.054045	-1.074712	-0.174930
C	-3.037778	-1.122933	0.196671
C	-3.021450	-3.558181	0.229901
C	4.468640	-3.476909	-0.191494
C	4.460582	-1.028780	-0.238119
C	-4.408518	-3.548067	0.303867
C	-4.444683	-1.099872	0.267407
H	5.031863	-4.412345	-0.212386
C	5.151749	-2.267071	-0.243093
H	-4.954699	-4.492140	0.359802
C	-5.113182	-2.349686	0.318585
C	5.173166	0.237481	-0.295126
C	-5.179547	0.154296	0.287578
C	-4.574853	1.361655	0.863278
C	-3.752982	1.299688	2.006013
C	-4.806165	2.630736	0.296381
C	-3.190695	2.447045	2.551226
C	-4.236391	3.775568	0.838616
H	-2.565903	2.369493	3.443670
H	-4.421657	4.744437	0.369613
C	-3.426226	3.691436	1.969719
C	4.552488	1.411694	-0.919773
C	3.742837	1.292263	-2.066756
C	4.754161	2.704954	-0.397767
C	4.168027	3.817962	-0.986880
C	3.164077	2.407794	-2.658734
H	4.330282	4.806698	-0.552008
H	2.549298	2.285539	-3.553136
C	3.370557	3.676992	-2.121389
C	-6.535560	0.230071	-0.269391
C	-7.521588	1.042800	0.324677
C	-6.898235	-0.493906	-1.422689
C	-8.804912	1.118463	-0.201641
C	-8.180170	-0.410792	-1.951538

Table S11 – continued

atom symbol	x (Å)	y (Å)	z (Å)
H	-9.552222	1.747260	0.287485
H	-8.428691	-0.973887	-2.853933
C	-9.142489	0.393160	-1.343075
C	6.520288	0.357484	0.275021
C	6.881593	-0.322221	1.455453
C	7.498617	1.169609	-0.332632
C	8.154609	-0.197398	1.997413
C	8.773189	1.287081	0.207207
H	8.401907	-0.726521	2.920448
H	9.514882	1.914266	-0.292448
C	9.109437	0.605355	1.375589
H	-7.273505	1.608139	1.224336
H	-10.150664	0.455935	-1.757903
H	-6.150148	-1.113166	-1.920160
H	-5.429555	2.709163	-0.595668
H	-6.200348	-2.367995	0.398390
H	-2.461206	-4.493991	0.215411
H	-2.479047	-0.189162	0.133481
H	-3.573028	0.334598	2.482026
H	-2.980662	4.592290	2.396757
H	2.912083	4.552884	-2.585194
H	3.585611	0.307058	-2.508410
H	5.366890	2.828125	0.496644
H	7.251876	1.700747	-1.253240
H	2.478304	-0.149579	-0.146858
H	2.538228	-4.454264	-0.080069
H	6.239595	-2.268235	-0.315817
H	6.138828	-0.939604	1.963106
H	10.110674	0.700942	1.800836

Table S12: Cartesian coordinates for C_2N_2-(trityl$^{\bullet}$)$_2$ optimized geometry in the first excited singlet state S_1 obtained at TD-DFT level (CAM-B3LYP/def2-SVP).

atom symbol	x (Å)	y (Å)	z (Å)
C	-0.845167	-2.195978	0.079711
N	0.019530	-1.181655	0.013985
N	0.061584	-3.300575	0.036938
C	0.928304	-2.287181	-0.029284
C	2.358950	-2.268053	-0.120255
C	-2.276801	-2.212786	0.168183
C	3.092050	-3.470019	-0.151434
C	3.050158	-1.051014	-0.177066
C	-3.010340	-1.020318	0.201147
C	-2.963470	-3.439446	0.220364
C	4.478439	-3.448863	-0.228588
C	4.452855	-1.016635	-0.252448
C	-4.351614	-3.468106	0.295209
C	-4.415406	-1.035626	0.273556
H	5.035663	-4.387461	-0.268956
C	5.166167	-2.239532	-0.265854
H	-4.874120	-4.425485	0.352947
C	-5.082153	-2.285115	0.309173
C	5.171657	0.242837	-0.347122
C	-5.177689	0.196188	0.339652
C	-4.637837	1.359307	1.020688
C	-3.779195	1.219139	2.135191
C	-4.949585	2.670644	0.592271
C	-3.271793	2.331279	2.789133
C	-4.425235	3.777751	1.240533
H	-2.621596	2.195857	3.655507
H	-4.662086	4.779710	0.876707
C	-3.587852	3.614806	2.344857
C	4.599028	1.359649	-1.074652
C	3.754805	1.147650	-2.189392
C	4.858642	2.696916	-0.692540
C	4.301196	3.759355	-1.386028
C	3.214419	2.215568	-2.888447
H	4.497820	4.781981	-1.057478
H	2.576477	2.024717	-3.753459
C	3.480725	3.525424	-2.490343
C	-6.492502	0.265956	-0.283103
C	-7.536540	1.028694	0.285832
C	-6.765971	-0.438571	-1.476018
C	-8.790219	1.075074	-0.304535
C	-8.018459	-0.374891	-2.070888

Table S12 – continued

atom symbol	x (Å)	y (Å)	z (Å)
H	-9.587831	1.654606	0.164899
H	-8.200918	-0.913217	-3.003197
C	-9.036767	0.377183	-1.487646
C	6.469238	0.378682	0.297388
C	6.746545	-0.290857	1.509968
C	7.495047	1.169189	-0.268019
C	7.983306	-0.165733	2.127363
C	8.733397	1.277098	0.345642
H	8.167875	-0.677613	3.074031
H	9.517504	1.877157	-0.120726
C	8.982873	0.614205	1.548372
H	-7.360347	1.558261	1.222902
H	-10.023145	0.420234	-1.954196
H	-5.968248	-1.011999	-1.948609
H	-5.580854	2.809414	-0.286004
H	-6.167791	-2.315031	0.402754
H	-2.370106	-4.355731	0.201783
H	-2.458556	-0.082255	0.135008
H	-3.536072	0.220918	2.498425
H	-3.178799	4.488519	2.856446
H	3.044913	4.364144	-3.037156
H	3.549180	0.128585	-2.515850
H	5.474203	2.891324	0.186282
H	7.318351	1.670375	-1.220435
H	2.462833	-0.133549	-0.131557
H	2.535707	-4.409021	-0.115048
H	6.252252	-2.231125	-0.359342
H	5.962393	-0.885248	1.979219
H	9.957199	0.705736	2.032890

Table S13: Cartesian coordinates for 5AP-(trityl$^{\bullet}$)$_2$ optimized geometry in the triplet ground state obtained at DFT level (UBHandHLYP/6-31++G(d,p)).

atom symbol	x (Å)	y (Å)	z (Å)
C	0.000000	-0.343167	0.000002
C	2.277033	-0.283931	0.060052
C	1.218403	1.725612	0.032992
C	0.000002	3.790614	-0.000009
C	-1.218401	1.725613	-0.032999
C	-2.277034	-0.283929	-0.060048
H	0.000002	4.868412	-0.000012
C	3.538658	-1.049064	0.092971
C	-3.538660	-1.049061	-0.092962
N	0.000000	1.059959	-0.000002
C	-4.757512	-0.380612	-0.110570
C	3.509845	-2.443667	0.100687
C	-3.509848	-2.443664	-0.100670
C	4.757512	-0.380615	0.110574
C	-5.914822	-2.481381	-0.130847
C	-4.702755	-3.148864	-0.121776
H	-4.687871	-4.226331	-0.137699
C	-5.972268	-1.077347	-0.127815
C	-7.249954	-0.369990	-0.140215
C	5.972267	-1.077351	0.127822
C	4.702752	-3.148867	0.121796
C	5.914819	-2.481385	0.130862
H	4.687867	-4.226334	0.137726
C	7.249954	-0.369994	0.140217
N	1.146576	-0.992037	0.029891
N	2.358689	1.031944	0.063379
N	-2.358689	1.031946	-0.063382
N	-1.146578	-0.992036	-0.029883
C	7.380369	0.889990	0.862749
C	6.669879	1.128209	2.051367
C	8.221675	1.914517	0.396386
C	8.341670	3.111253	1.079874
C	6.799000	2.322611	2.737419
H	8.984826	3.883587	0.689905
H	6.250416	2.470615	3.653514
C	7.633760	3.323531	2.256469
C	-7.380360	0.890005	-0.862730
C	-6.669831	1.128252	-2.051320
C	-8.221694	1.914512	-0.396377
C	-6.798943	2.322664	-2.737356
C	-8.341681	3.111259	-1.079850

Table S13 – continued

atom symbol	x (Å)	y (Å)	z (Å)
H	-6.250330	2.470691	-3.653429
H	-8.984861	3.883578	-0.689890
C	-7.633733	3.323566	-2.256417
C	8.395424	-0.928830	-0.571361
C	8.233515	-1.626681	-1.779805
C	9.701879	-0.792009	-0.073599
C	10.785538	-1.328502	-0.746167
C	9.320100	-2.153647	-2.455163
H	9.165726	-2.672191	-3.387593
H	11.774940	-1.219288	-0.332255
C	10.603405	-2.010810	-1.942561
C	-8.395433	-0.928836	0.571340
C	-8.233536	-1.626729	1.779760
C	-9.701884	-0.791980	0.073578
C	-9.320130	-2.153704	2.455096
C	-10.785552	-1.328481	0.746123
H	-9.165766	-2.672284	3.387508
H	-11.774951	-1.219240	0.332212
C	-10.603432	-2.010833	1.942494
H	2.561280	-2.949756	0.090501
H	4.753459	0.693878	0.091708
H	-2.561283	-2.949754	-0.090480
H	-4.753460	0.693882	-0.091713
H	7.246726	-1.735169	-2.197190
H	11.448567	-2.424996	-2.467361
H	9.858476	-0.276722	0.858830
H	6.831542	-3.045688	0.158547
H	7.731117	4.254679	2.790165
H	6.027612	0.358858	2.444891
H	8.766812	1.771220	-0.521022
H	-7.731083	4.254722	-2.790100
H	-8.766862	1.771191	0.521008
H	-6.027541	0.358915	-2.444834
H	-9.858470	-0.276656	-0.858834
H	-7.246749	-1.735245	2.197143
H	-11.448600	-2.425026	2.467278
H	-6.831545	-3.045682	-0.158527
C	-1.204287	3.115330	-0.033305
C	1.204290	3.115329	0.033290
H	2.153134	3.617043	0.059641
H	-2.153130	3.617045	-0.059658

Table S14: Cartesian coordinates for 5AP-(trityl$^{\bullet}$)$_2$ optimized geometry in the first excited triplet state T_1 obtained at TD-DFT level (CAM-B3LYP/def2-SVP).

atom symbol	x (Å)	y (Å)	z (Å)
C	0.000001	-0.417638	-0.000012
C	2.295414	-0.356303	0.058875
C	1.233115	1.678621	0.032047
C	0.000005	3.787057	-0.000032
C	-1.233109	1.678624	-0.032087
C	-2.295412	-0.356298	-0.058898
H	0.000007	4.877295	-0.000038
C	3.565324	-1.095444	0.090898
C	-3.565323	-1.095437	-0.090913
N	0.000002	1.005630	-0.000015
C	-4.784046	-0.410312	-0.108411
C	3.563530	-2.499753	0.094776
C	-3.563532	-2.499747	-0.094790
C	4.784047	-0.410321	0.108403
C	-5.979801	-2.504057	-0.119747
C	-4.770277	-3.188759	-0.111531
H	-4.769616	-4.280915	-0.125085
C	-6.015649	-1.092303	-0.120342
C	-7.283649	-0.365256	-0.128465
C	6.015650	-1.092313	0.120340
C	4.770275	-3.188768	0.111523
C	5.979800	-2.504067	0.119744
H	4.769612	-4.280923	0.125077
C	7.283650	-0.365266	0.128474
N	1.153915	-1.052303	0.029415
N	2.351734	1.000359	0.060886
N	-2.351730	1.000363	-0.060921
N	-1.153915	-1.052300	-0.029436
C	7.392665	0.923973	0.814053
C	6.670863	1.193086	1.995544
C	8.230598	1.945822	0.320672
C	8.336665	3.168461	0.970712
C	6.784643	2.414192	2.647384
H	8.985421	3.943980	0.557188
H	6.223274	2.587145	3.568412
C	7.616323	3.411018	2.139391
C	-7.392664	0.923995	-0.814022
C	-6.670858	1.193132	-1.995505
C	-8.230602	1.945833	-0.320626
C	-6.784639	2.414249	-2.647323
C	-8.336671	3.168483	-0.970644

Table S14 – continued

atom symbol	x (Å)	y (Å)	z (Å)
H	-6.223267	2.587221	-3.568346
H	-8.985431	3.943992	-0.557110
C	-7.616325	3.411063	-2.139316
C	8.453493	-0.928482	-0.552200
C	8.322403	-1.656629	-1.752520
C	9.754030	-0.763021	-0.033313
C	10.860684	-1.301892	-0.677050
C	9.431608	-2.187515	-2.398437
H	9.299293	-2.736060	-3.333748
H	11.854300	-1.168756	-0.243181
C	10.707811	-2.016033	-1.864444
C	-8.453495	-0.928485	0.552195
C	-8.322407	-1.656656	1.752500
C	-9.754030	-0.763012	0.033308
C	-9.431614	-2.187554	2.398404
C	-10.860686	-1.301895	0.677032
H	-9.299302	-2.736119	3.333705
H	-11.854301	-1.168750	0.243162
C	-10.707816	-2.016060	1.864412
H	2.608988	-3.025498	0.084293
H	4.764937	0.678115	0.089628
H	-2.608990	-3.025492	-0.084311
H	-4.764933	0.678124	-0.089637
H	7.331499	-1.786453	-2.190301
H	11.578914	-2.436482	-2.371344
H	9.888010	-0.218624	0.902735
H	6.916349	-3.062351	0.148161
H	7.703752	4.371149	2.652347
H	6.028249	0.417798	2.415109
H	8.790030	1.776045	-0.600468
H	-7.703755	4.371203	-2.652255
H	-8.790038	1.776036	0.600508
H	-6.028240	0.417853	-2.415081
H	-9.888006	-0.218597	-0.902729
H	-7.331504	-1.786491	2.190281
H	-11.578921	-2.436518	2.371301
H	-6.916351	-3.062340	-0.148160
C	-1.206070	3.089743	-0.031718
C	1.206079	3.089741	0.031662
H	2.173095	3.590754	0.057200
H	-2.173084	3.590759	-0.057263

Table S15: Cartesian coordinates for 5AP-(trityl•)$_2$ optimized geometry in the first excited singlet state S_1 obtained at TD-DFT level (CAM-B3LYP/def2-SVP).

atom symbol	x (Å)	y (Å)	z (Å)
C	-0.000001	-0.385059	0.000026
C	2.295334	-0.333631	0.064496
C	1.235732	1.705410	0.035618
C	0.000001	3.801895	-0.000050
C	-1.235732	1.705410	-0.035642
C	-2.295336	-0.333631	-0.064449
H	0.000001	4.893156	-0.000070
C	3.561378	-1.083184	0.099210
C	-3.561379	-1.083186	-0.099139
N	-0.000000	1.040006	0.000002
C	-4.778910	-0.403438	-0.109142
C	3.550458	-2.488027	0.108598
C	-3.550461	-2.488030	-0.108470
C	4.778909	-0.403437	0.109176
C	-5.966963	-2.504633	-0.125078
C	-4.756297	-3.181749	-0.124005
H	-4.750989	-4.273798	-0.146364
C	-6.014653	-1.088348	-0.119619
C	-7.276232	-0.370990	-0.121408
C	6.014652	-1.088346	0.119668
C	4.756294	-3.181746	0.124151
C	5.966961	-2.504632	0.125184
H	4.750986	-4.273794	0.146557
C	7.276233	-0.370988	0.121415
N	1.153837	-1.025465	0.032452
N	2.352616	1.021041	0.067427
N	-2.352617	1.021040	-0.067429
N	-1.153838	-1.025466	-0.032378
C	7.382333	0.926624	0.769152
C	6.622183	1.248198	1.919119
C	8.255678	1.927249	0.278972
C	8.356171	3.165020	0.895393
C	6.732319	2.485208	2.536078
H	9.028676	3.919299	0.480330
H	6.143041	2.692764	3.432269
C	7.597607	3.455519	2.030071
C	-7.382303	0.926635	-0.769120
C	-6.622040	1.248264	-1.918997
C	-8.255732	1.927219	-0.279005
C	-6.732147	2.485289	-2.535932
C	-8.356198	3.165003	-0.895403

Table S15 – continued

atom symbol	x (Å)	y (Å)	z (Å)
H	-6.142778	2.692888	-3.432052
H	-9.028773	3.919250	-0.480392
C	-7.597519	3.455558	-2.029990
C	8.442911	-0.953032	-0.528994
C	8.321360	-1.739866	-1.698274
C	9.748362	-0.757035	-0.020716
C	10.856307	-1.317491	-0.638823
C	9.433362	-2.291242	-2.317617
H	9.304910	-2.880066	-3.228755
H	11.848496	-1.160829	-0.209392
C	10.709762	-2.087514	-1.793029
C	-8.442939	-0.953049	0.528933
C	-8.321434	-1.739971	1.698158
C	-9.748374	-0.756977	0.020642
C	-9.433465	-2.291362	2.317437
C	-10.856347	-1.317447	0.638685
H	-9.305048	-2.880258	3.228533
H	-11.848523	-1.160724	0.209246
C	-10.709848	-2.087559	1.792837
H	2.594312	-3.010661	0.103511
H	4.760151	0.684430	0.074489
H	-2.594315	-3.010664	-0.103354
H	-4.760152	0.684431	-0.074505
H	7.333804	-1.888661	-2.136905
H	11.583656	-2.525013	-2.280030
H	9.879588	-0.178107	0.894432
H	6.899390	-3.068563	0.164067
H	7.681239	4.429500	2.516554
H	5.960167	0.493873	2.345818
H	8.837976	1.727549	-0.621379
H	-7.681129	4.429551	-2.516455
H	-8.838125	1.727472	0.621275
H	-5.959950	0.493972	-2.345642
H	-9.879564	-0.177973	-0.894464
H	-7.333890	-1.888831	2.136795
H	-11.583765	-2.525070	2.279788
H	-6.899393	-3.068565	-0.163942
C	-1.209231	3.115395	-0.035503
C	1.209232	3.115395	0.035427
H	2.172403	3.622631	0.063590
H	-2.172401	3.622631	-0.063686

Table S16: Cartesian coordinates for C_2N_2-(allyl$^{\bullet}$)$_2$ optimized geometry in the triplet ground state obtained at DFT level (UBHandHLYP/6-31++G(d,p)).

atom symbol	x (Å)	y (Å)	z (Å)
C	-0.904267	-0.057176	0.000024
N	0.070444	1.050094	0.000014
N	-0.070445	-1.050102	0.000008
C	0.904269	0.057167	0.000004
C	2.347208	-0.010974	-0.000006
C	-2.347209	0.010974	0.000003
C	2.916958	-1.273129	0.000087
H	3.986784	-1.393391	0.000102
C	3.062406	1.186171	-0.000099
H	2.548009	2.130616	-0.000137
C	-2.916953	1.273134	0.000080
H	-3.986780	1.393399	0.000034
C	-3.062410	-1.186164	-0.000094
H	-2.548013	-2.130610	-0.000178
H	-4.138580	-1.180993	-0.000106
H	4.138576	1.181003	-0.000127
H	2.298475	-2.152704	0.000116
H	-2.298472	2.152710	0.000154

References

(1) Ohno, K. Some remarks on the Pariser-Parr-Pople method. *Theoretica chimica acta* **1964**, *2*, 219–227.

(2) Albert, I. D. L.; Ramasesha, S.; Das, P. K. Properties of some low-lying electronic states in polymethineimines and poly(2,3-diazabutadienes). *Physical Review B* **1991**, *43*, 7013–7019.

(3) Thomas, S.; Pati, Y.; Ramasesha, S. Linear and nonlinear optical properties of expanded porphyrins: A DMRG study. *The Journal of Physical Chemistry A* **2013**, *117*, 7804–7809.

(4) Lehoucq, R.; Sorensen, D.; Yang, C. *ARPACK Users' Guide*; Society for Industrial and Applied Mathematics, 1998.

(5) Lakowicz, J. R. *Principles of fluorescence spectroscopy*, 3rd ed.; Springer: New York, 2006.

(6) Pariser, R.; Parr, R. G. A Semi-Empirical Theory of the Electronic Spectra and Electronic Structure of Complex Unsaturated Molecules. II. *The Journal of Chemical Physics* **1953**, *21*, 767–776.

(7) Soos, Z. G.; Ramasesha, S. Valence-bond theory of linear Hubbard and Pariser-Parr-Pople models. *Physical Review B* **1984**, *29*, 5410–5422.

(8) Prodhan, S.; Soos, Z. G.; Ramasesha, S. Model for triplet state engineering in organic light emitting diodes. *The Journal of Chemical Physics* **2014**, *140*, 214313.

(9) Favini, G.; Vandoni, I.; Simonetta, M. Calculation of electronic spectra of aza-benzenes and aza-naphthalenes by the Pariser-Parr-Pople method. *Theoretica Chimica Acta* **1965**, *3*, 45–58.

(10) Nishimoto, K.; Forster, L. S. SCFMO calculations of heteroatomic systems with the variable? approximation. *Theoretica Chimica Acta* **1966**, *4*, 155–165.

(11) Michl, J.; Koutecky, J.; Becker, R. S.; Earhart, C. E. A note on the parameters for heteroatoms in Pariser-Parr-Pople (PPP) calculations. *Theoretica Chimica Acta* **1970**, *19*, 92–97.

(12) Hinze, J.; Beveridge, D. L. Parametrization of semiempirical π-electron molecular orbital calculations. π Systems containing carbon, nitrogen, oxygen, and fluorine. *Journal of the American Chemical Society* **1971**, *93*, 3107–3114.

(13) Zahradník, R.; Tesařová, I.; Pancíř, J. Experimental and theoretical (HMO and LCI-SCF) study of singlet-triplet transitions in conjugated hydrocarbons and their derivatives. *Collection of Czechoslovak Chemical Communications* **1971**, *36*, 2867–2880.

(14) Griffiths, J. Practical aspects of colour prediction of organic dye molecules. *Dyes and Pigments* **1982**, *3*, 211–233.

(15) Grossjean, M. F.; Tavan, P. Wavelength regulation in bacteriorhodopsin and halorhodopsin: A Pariser–Parr–Pople multireference double excitation configuration interaction study of retinal dyes. *The Journal of Chemical Physics* **1988**, *88*, 4884–4896.

(16) Albert, I. D. L.; Das, P. K.; Ramasesha, S. Optical nonlinearities in symmetric cyanine dyes and related systems. *Journal of the Optical Society of America B* **1993**, *10*, 1365.

(17) Mukhopadhyay, S.; Topham, B. J.; Soos, Z. G.; Ramasesha, S. Neutral and Charged Excited States in Polar Organic Films: Origin of Unusual Electroluminescence in Tri-*p*-tolylamine-Based Hole Conductors. *The Journal of Physical Chemistry A* **2008**, *112*, 7271–7279.

(18) Kumar, M.; Pati, Y. A.; Ramasesha, S. A density matrix renormalization group method study of optical properties of porphines and metalloporphines. *The Journal of Chemical Physics* **2012**, *136*, 014112.

(19) Bedogni, M.; Giavazzi, D.; Di Maiolo, F.; Painelli, A. Shining Light on Inverted Singlet–Triplet Emitters. *Journal of Chemical Theory and Computation* **2023**, *20*, 902–913.

(20) Bedogni, M.; Di Maiolo, F. Singlet–Triplet Inversion in Triangular Boron Carbon Nitrides. *Journal of Chemical Theory and Computation* **2024**, DOI:10.1021/acs.jctc.4c00706.

(21) Dubbini, M.; Bonvini, F.; Savi, L.; Di Maiolo, F. Turning on Organic Radical Emitters. *The Journal of Physical Chemistry C* **2024**, *128*, 18158–18169.

(22) Sandoval-Salinas, M. E.; Carreras, A.; Casanova, D. Triangular graphene nanofragments: open-shell character and doping. *Physical Chemistry Chemical Physics* **2019**, *21*, 9069–9076.

(23) Casanova, D. Restricted active space configuration interaction methods for strong correlation: Recent developments. *WIREs Computational Molecular Science* **2022**, *12*, e1561.

(24) Casanova, D.; Head-Gordon, M. Restricted active space spin-flip configuration interaction approach: theory, implementation and examples. *Physical Chemistry Chemical Physics* **2009**, *11*, 9779.

(25) Nakano, M.; Champagne, B. Theoretical Design of Open-Shell Singlet Molecular Systems for Nonlinear Optics. *The Journal of Physical Chemistry Letters* **2015**, *6*, 3236–3256.

(26) Nakano, M.; Champagne, B. Nonlinear optical properties in open-shell molecular systems. *WIREs Computational Molecular Science* **2016**, *6*, 198–210.

(27) Frisch, M. J. et al. Gaussian~16 Revision B.01. 2016; Gaussian Inc. Wallingford CT.

(28) Neese, F. The ORCA program system. *WIRES Comput. Molec. Sci.* **2012**, *2*, 73–78.

(29) Franz, M.; Neese, F.; Richert, S. Calculation of exchange couplings in the electronically excited state of molecular three-spin systems. *Chemical Science* **2022**, *13*, 12358–12366.

(30) Poh, Y. R.; Morozov, D.; Kazmierczak, N. P.; Hadt, R. G.; Groenhof, G.; Yuen-Zhou, J. Alternant Hydrocarbon Diradicals as Optically Addressable Molecular Qubits. *Journal of the American Chemical Society* **2024**, *146*, 15549–15561.
